# Supplementary material for: Complete Chloroplast Genome Determination of Ranunculus sceleratus from Republic of Korea (Ranunculaceae) and Comparative Chloroplast Genomes of the Members of the Ranunculus Genus
Source: Genes (Basel). 2023 May 25;14(6):1149. doi: 10.3390/genes14061149 (PMC10298533; doi:10.3390/genes14061149)

Table S1. List of species information used for comparative chloroplast genome of *Ranunculus*

No.	Science name	GenBank accession number	Reference (location)
1	*Ranunculus austro-oreganus*	KX639503	Unpublished
2	*Ranunculus bungei*	MK253468	He et al. 2019<br>(Qinghai, China)
3	*Ranunculus cantoniensis*	NC_045920	He et al. 2019<br>(MangShan, China)
4	*Ranunculus japonicus*	MZ169045	Zeng et al. 2021<br>(Xuancheng, China)
5	*Ranunculus macranthus*	DQ359689	Raubeso et al. 2007<br>(Texas, United States of America)
6	*Ranunculus occidentalis*	NC_031651	Unpublished
7	*Ranunculus pekinensis*	OK166810	Unpublished<br>(China)
8	*Ranunculus reptans*	NC_036977	Dann et al. 2017<br>(Germany)
9	*Ranunculus repens*	NC_036976	Dann et al. 2017<br>(Germany)
10	*Ranunculus sceleratus*	MK253452	He et al. 2019<br>(Beijing, China)
11	*Ranunculus sceleratus*	ON755204	This study (Chungcheongnam-do,<br>Korea)
12	*Ranunculus yunnanensis*	MZ703201	Rao et al. 2022

Table S2. List of genes in the Ranunculus species.

Gene category		Gene name
Photosynthesis	Photosystem I	*psaA*, *B*, *C*, *I*, *J*
	Photosystem II	*psbA*, *B*, *C*, *D*, *E*, *F*, *H*, *I*, *J*, *K*, *L*, *M*, *N*, *T*, *Z*
	Cytochrome b/f complex	*petA*, *B*, *D*, *G*, *L*, *N*
	ATP synthase	*atpA*, *B*, *E*, *F*, *H*, *I*
	NADH dehydrogenase	*ndhA*, *B* (×2), *C*, *D*, *E*, *F*, *G*, *H*, *I*, *J*, *K*
	Rubisco	*rbcL*
Self-replication	Transfer RNAs	*trnA-UGC* (×2), *C-GCA*, *D-GUC*, *E-UUC*, *F-GAA*, *trnfM-CAU*, *G-GCC*, *G-UCC*, *H-GUG*, *I-CAU* (×2), *I-GAU* (×2), *K-UUU*, *L-CAA* (×2), *L-UAA*, *L-UAG*, *M-CAU*, *N-GUU* (×2), *P-UGG*, *Q-UGG*, *R-ACG* (×2), *R-UCU*, *S-GCU*, *S-GGA*, *S-UGA*, *T-GUU*, *T-UGU*, *V-GAC* (×2), *V-UAC*, *W-CCA*, *Y-GUA*
	Ribosomal RNAs	*rrn 16* (×2), *23* (×2), *4.5* (×2), *5* (×2)
	RNA polymerase subunits	*rpoA*, *rpoB*, *rpoC1*, *rpoC2*
	Large subunit ribosomal proteins	*rpl2* (×2), *14*, *16*, *20*, *22*, *23*, *32*, *33*, *36*
	Small subunit ribosomal proteins	*rps2*, *3*, *4*, *7* (×2), *8*, *11*, *12* (×2), *14*, *15*, *16*, *18*, *19* (×2)
Other genes	Maturase	*matK*
	Envelope membrane protein	*cemA*
	Subunit of acetyl-CoA-carboxylase	*accD*
	Proteins of unknown function	*ycf1* (×2), *ycf2* (×2), *ycf3*, *ycf4*
	c-type cytochrome synthesis gene	*ccsA*
	Pseudogenes	***Ψ infA*, *Ψ ycf15***

Bold text indicates genes only present in certain species (*Ψ infA*: *R. sceleratus* ON755204, *R. sceleratus* MK253452; *Ψ ycf15*: *R. austro-oreganus* KX639503; *Ψ ycf1*: *R. cantoniensis, R. macranthus, and R. reptans*). Pseudogenes are indicated by *ψ*.

Table S3. Comparative information of the cp genome structure of the species *Ranunculus* genus

Feature	*Ranunculus austro-oreganus*	*Ranunculus bungei*	*Ranunculus cantoniensis*
Total genome size (bp)	154,493	156,082	155,154
Large single copy (bp)	83,582	85,436	84,562
Small single copy (bp)	21,249	19,942	18,879
Inverted repeat region (bp)	24,831	25,352	25,838
GC content (%)			
Total genome	37.8	37.8	37.9
LSC	35.9	36.0	36.0
SSC	31.5	31.3	31.1
IR	43.6	43.5	43.5
Genes number			
Total number of genes	112	112	112
Protein-coding genes	78	78	78
tRNA genes	30	30	30
rRNA genes	4	4	4
Number of Protein-coding genes in IR	5	6	6

Feature	*Ranunculus japonicus*	*Ranunculus macranthus*	*Ranunculus occidentalis*
Total genome size (bp)	156,981	155,129	154,474
Large single copy (bp)	85,454	84,636	83,543
Small single copy (bp)	18,897	18,909	21,269
Inverted repeat region (bp)	26,315	25,792	24,831
GC content (%)			
Total genome	37.7	37.9	37.8
LSC	36.0	36.0	35.9
SSC	31.3	31.0	31.6
IR	42.9	43.5	43.6
Genes number			
Total number of genes	112	112	112
Protein-coding genes	78	78	78
tRNA genes	30	30	30
rRNA genes	4	4	4
Number of Protein-coding genes in IR	6	6	5

Feature	*Ranunculus pekinensis*	*Ranunculus reptans*	*Ranunculus repens*
Total genome size (bp)	156,139	157,239	154,247
Large single copy (bp)	85,431	85,458	84,225
Small single copy (bp)	19,974	17,667	18,434
Inverted repeat region (bp)	25,367	27,057	25,794
GC content (%)			
Total genome	37.8	37.9	37.9
LSC	36.0	36.2	36.0
SSC	31.3	31.3	31.1
IR	43.5	42.8	43.5
Genes number			
Total number of genes	112	112	112
Protein-coding genes	78	78	78
tRNA genes	30	30	30
rRNA genes	4	4	4
Number of Protein-coding genes in IR	6	7	6

Feature	*Ranunculus yunnanensis*	*Ranunculus sceleratus* (China)	*Ranunculus sceleratus* (Korea)
Total genome size (bp)	156,050	156,324	156,329
Large single copy (bp)	85,556	85,835	85,840
Small single copy (bp)	19,772	19,885	19,885
Inverted repeat region (bp)	25,361	25,302	25,302
GC content (%)			
Total genome	37.9	37.9	37.9
LSC	36.1	36.1	36.1
SSC	31.5	31.7	31.7
IR	43.5	43.6	43.6
Genes number			
Total number of genes	112	112	112
Protein-coding genes	78	78	78
tRNA genes	30	30	30
rRNA genes	4	4	4
Number of Protein-coding genes in IR	6	6	6

Table S4. Repeat type information of *Ranunculus* species

Species	Forward	Reverse	Complementary	Palindromic	Total
R. sceleratus	11	10	7	3	31
R. sceleratus	11	10	7	3	31
R. austro-oreganus	11	10	8	2	31
R. bungei	12	11	8	3	34
R. cantoniensis	9	8	6	2	25
R. japonicus	24	20	18	2	64
R. macranthus	12	7	6	1	26
R. occidentalis	11	10	7	3	31
R. pekinensis	16	16	12	4	48
R. repens	13	8	6	2	29
R. reptans	12	11	7	4	34
R. yunnanensis	6	6	4	2	18
Total	148	127	96	31	402

Table S5. Distribution by species according to the long repeat sequence

Repeat length	*R. sceleratus*	*R. sceleratus*	*R. austro-oreganus*	*R. bungei*	*R. cantoniensis*	*R. japonicus*	*R. macranthus*	*R. occidentalis*	*R. pekinensis*	*R. repens*	*R. reptans*	*R. yunnanensis*
30	2	2	2	2	4	6	4	2	6	5	4	2
31	4	4	0	6	3	6	3	0	9	3	4	2
32	2	2	4	4	2	5	0	4	4	2	6	2
33	0	0	0	0	0	4	0	0	0	0	0	0
36	0	4	2	0	0	4	0	2	3	0	2	2
38	4	0	0	0	2	0	2	0	0	2	0	0
39	3	3	3	3	0	3	0	3	0	0	3	0
40	0	0	0	4	0	0	0	0	4	0	0	0
41	0	0	0	0	0	6	2	0	0	0	0	0
42	4	4	6	4	6	6	8	6	4	8	4	4
44	2	2	0	0	0	0	0	0	0	0	0	0
46	0	0	0	0	0	0	0	0	2	0	0	0
48	0	0	0	0	0	4	0	0	0	0	0	0
61	0	0	2	0	0	0	0	0	0	0	0	0
63	0	0	0	0	0	0	0	2	0	0	0	0
64	0	0	2	0	0	0	0	0	0	0	0	0
65	0	0	0	0	0	0	0	2	0	0	0	0

Table S6. SSR region information of the species cp genome of the genus *Ranunculus*

Species	Region	Mono-	Di-	Tri-	Tetra-	Penta-	Hexa-	Total
Ranunculus sceleratus (Korea)	LSC	21	5	2	4	2	0	34
	SSC	12	0	1	2	0	0	15
	IR	0	2	0	0	2	0	4
	Total	33	7	3	6	4	0	53
Ranunculus sceleratus (China)	LSC	21	5	2	4	1	0	33
	SSC	12	0	1	2	0	0	15
	IR	0	2	0	0	2	0	4
	Total	33	7	3	6	3	0	52
Ranunculus austro-oreganus	LSC	16	6	4	6	0	0	32
	SSC	6	1	0	2	0	0	9
	IR	2	2	0	2	0	0	6
	Total	24	9	4	10	0	0	47
Ranunculus bungei	LSC	18	7	1	4	1	0	31
	SSC	5	0	1	2	0	0	8
	IR	2	2	0	0	0	0	4
	Total	25	9	2	6	1	0	43

Ranunculus cantoniensis	LSC	14	7	1	4	1	0	27
	SSC	9	1	0	4	0	0	14
	IR	4	0	0	0	0	0	4
	Total	27	8	1	8	1	0	45
Ranunculus japonicus	LSC	19	5	4	6	0	0	34
	SSC	5	1	0	2	0	1	9
	IR	0	2	0	2	0	0	4
	Total	24	8	4	10	0	1	47
Ranunculus macranthus	LSC	16	7	3	2	1	0	29
	SSC	8	1	0	4	1	1	15
	IR	2	0	0	2	0	0	4
	Total	26	8	3	8	2	1	48
Ranunculus occidentalis	LSC	15	6	4	6	0	0	31
	SSC	5	1	0	2	0	0	8
	IR	2	2	0	2	0	0	6
	Total	22	9	4	10	0	0	45
Ranunculus pekinensis	LSC	19	7	1	4	0	0	31
	SSC	6	0	2	2	0	0	10
	IR	4	4	0	0	0	0	8
	Total	29	11	3	6	0	0	49

Ranunculus reptans	LSC	19	4	2	3	0	0	28
	SSC	2	3	0	3	0	0	8
	IR	2	0	0	0	0	0	2
	Total	23	7	2	6	0	0	38
Ranunculus repens	LSC	17	6	2	2	2	0	29
	SSC	9	1	0	4	0	0	14
	IR	1	0	0	0	0	0	1
	Total	27	7	2	6	2	0	44
Ranunculus yunnanensis	LSC	18	4	1	3	0	0	26
	SSC	8	1	1	1	0	0	11
	IR	0	0	0	0	0	0	0
	Total	26	5	2	4	0	0	37
Total SSR motifs		319 (58.21%)	95 (17.34%)	33 (6.02%)	86 (15.69%)	13 (2.37%)	2 (0.36%)	548 (100%)

Table S7. Sequence and tree-based species identification rate of combinatorial markers of the genus *Ranunculus*

No.	Region name	Alignment length (bp)	ML tree (%)	BLAST (%)	Total identify rate (%)
1	*petG-trnW-CCA* + *rps8-rpl14*	536	83.3	100	91.7
2	*petG-trnW-CCA* + *trnT-UGU-trnL-UAA* + *rps8-rpl14*	1244	100	100	100.0
3	*petG-trnW-CCA* + *trnT-UGU-trnL-UAA*	1602	83.3	100	91.7
4	*petG-trnW-CCA* + *trnK-UUU-rps16* + *rps8-rpl14*	947	91.7	100	95.9
5	*petG-trnW-CCA* + *trnK-UUU-rps16*	1305	83.3	100	91.7
6	*rpl32-trnL-UAG* + *rps8-rpl14*	576	91.7	100	95.9
7	*rpl32-trnL-UAG* + *trnT-UGU-trnL-UAA* + *rps8-rpl14*	2011	91.7	100	95.9
8	*rpl32-trnL-UAG* + *trnT-UGU-trnL-UAA*	2369	83.3	100	91.7
9	*rpl32-trnL-UAG* + *trnK-UUU-rps16* + *rps8-rpl14*	1714	91.7	100	95.9
10	*rpl32-trnL-UAG* + *trnK-UUU-rps16*	2072	83.3	100	91.7
11	*rpl16-rps3* + *rps8-rpl14*	576	100	100	100.0
12	*rpl16-rps3* + *trnT-UGU-trnL-UAA* + *rps8-rpl14*	1284	100	100	100.0
13	*rpl16-rps3* + *trnT-UGU-trnL-UAA*	1642	83.3	100	91.7
14	*rpl16-rps3* + *trnK-UUU-rps16* + *rps8-rpl14*	987	100	100	100.0
15	*rpl16-rps3* + *trnK-UUU-rps16*	1345	83.3	100	91.7
16	*rps16-trnQ-UUG* + *rps8-rpl14*	1889	100	100	100.0
17	*rps16-trnQ-UUG* + *trnT-UGU-trnL-UAA* + *rps8-rpl14*	2597	83.3	100	91.7
18	*rps16-trnQ-UUG* + *trnT-UGU-trnL-UAA*	2955	83.3	100	91.7
19	*rps16-trnQ-UUG* + *trnK-UUU-rps16* + *rps8-rpl14*	2300	91.7	100	95.9
20	*rps16-trnQ-UUG* + *trnK-UUU-rps16*	2658	83.3	100	91.7
21	*accD-psaI* + *rps8-rpl14*	1185	100	100	100.0
22	*accD-psaI* + *trnT-UGU-trnL-UAA* + *rps8-rpl14*	1893	100	100	100.0
23	*accD-psaI* + *trnT-UGU-trnL-UAA*	2251	83.3	100	91.7

24	*accD-psaI + trnK-UUU-rps16 + rps8-rpl14*	1596	100	100	100.0
25	*accD-psaI + trnK-UUU-rps16*	1954	83.3	100	91.7
26	*trnG-GCC-trnfM-CAU + rps8-rpl14*	576	100	100	100.0
27	*trnG-GCC-trnfM-CAU + trnT-UGU-trnL-UAA + rps8-rpl14*	1284	100	100	100.0
28	*trnG-GCC-trnfM-CAU + trnT-UGU-trnL-UAA*	1642	83.3	100	91.7
29	*trnG-GCC-trnfM-CAU + trnK-UUU-rps16 + rps8-rpl14*	987	100	100	100.0
30	*trnG-GCC-trnfM-CAU + trnK-UUU-rps16*	1345	83.3	100	91.7
31	*trnT-UGU-trnL-UAA + rps8-rpl14*	1424	100	100	100.0
32	*psbZ-trnG-GCC + rps8-rpl14*	784	100	100	100.0
33	*psbZ-trnG-GCC + trnT-UGU-trnL-UAA + rps8-rpl14*	1492	100	100	100.0
34	*psbZ-trnG-GCC + trnT-UGU-trnL-UAA*	1850	91.7	100	95.9
35	*psbZ-trnG-GCC + trnK-UUU-rps16 + rps8-rpl14*	1195	100	100	100.0
36	*psbZ-trnG-GCC + trnK-UUU-rps16*	1553	91.7	100	95.9
37	*trnK-UUU-rps16 + rps8-rpl14*	1127	100	100	100.0

Table S8. The potential positive selection test based on the branch-site model of *Ranunculus*

Gene name	Alternative hypothesis			Null hypothesis			2Δl	df	p-value	Positive selection site	
	InL	np	ω>1	InL	np	ω=1				BEB	NEB
accD	-3915.3891	40	1.00000	-3915.3924	39	1.00000	0.00663	1	0.06491	28 T 0.532	
atpA	-3398.5573	40	1.00000	-3398.5573	39	1.00000	0.00000	1	1.00000		
atpB	-3330.0126	40	1.00000	-3330.0126	39	1.00000	0.00000	1	1.00000		
atpE	-849.7646	40	1.00000	-851.1009	39	1.00000	2.67267	1	0.89792		
atpF	-1256.4586	40	71.39778	-1257.7742	39	1.00000	2.63116	1	0.89522	**3 K 0.724 90 A 0.708**	**90 A 0.950****
atpH	-466.6377	40	1.00000	-466.6377	39	1.00000	0.00000	1	1.00000		
atpI	-1540.1570	40	1.00000	-1540.1909	39	1.00000	0.06777	1	0.20539	55 I 0.600	
ccsA	-3417.9233	40	1.00000	-3417.9233	39	1.00000	0.00000	1	1.00000	86 Y 0.551 274 K 0.535	
cemA	-1831.5027	40	20.25288	-1831.5382	39	1.00000	0.07100	1	0.21012	148 C 0.504	
clpP	-1928.8208	40	1.00000	-1928.8208	39	1.00000	0.00000	1	1.00000		
matK	-4914.1306	40	1.00000	-4914.1306	39	1.00000	0.00000	1	1.00000		
ndhA	-2981.7773	40	1.00000	-2981.7773	39	1.00000	0.00000	1	1.00000		
ndhB	-2424.4095	40	1.00000	-2424.4211	39	1.00000	0.02331	1	0.12134		
ndhC	-798.8465	40	1.00000	-798.8465	39	1.00000	0.00000	1	1.00000		
ndhE	-739.6977	40	50.14148	-739.8330	39	1.00000	0.27066	1	0.39711	**68 G 0.932***	
ndhF	-4576.0317	40	3.34360	-4576.4050	39	1.00000	0.74655	1	0.61243	**43 F 0.927* 302 L 0.512**	**43 F 0.926***
ndhG	-1492.3836	40	1.00000	-1492.3836	39	1.00000	0.00000	1	1.00000	51 T 0.545 75 I 0.530	
ndhH	-2838.6940	40	1.00000	-2838.6940	39	1.00000	0.00000	1	1.00000	342 V 0.513	
ndhI	-1433.2738	40	1.00000	-1433.2738	39	1.00000	0.00000	1	1.00000		
ndhJ	-1019.2000	40	1.00000	-1019.2000	39	1.00000	0.00000	1	1.00000		
ndhK	-1524.0873	40	1.95628	-1524.0980	39	1.00000	0.02136	1	0.11620	203 I 0.616	
petA	-2289.8385	40	26.44419	-2289.9266	39	1.00000	0.17623	1	0.32537	31 Q 0.617 124 N 0.639	

										238 R 0.620
petB	-1353.5120	40	1.00000	-1353.5120	39	1.00000	0.00000	1	1.00000	
petD	-1052.7162	40	1.00000	-1052.7162	39	1.00000	0.00000	1	1.00000	
petL	-177.7136	40	1.00000	-177.7136	39	1.00000	0.00000	1	1.00000	
petN	-122.4277	40	3.13740	-122.4277	39	1.00000	0.00000	1	1.00000	
psaA	-4462.7022	40	1.00000	-4462.7022	39	1.00000	0.00000	1	1.00000	
psaB	-4286.3863	40	1.00000	-4286.3863	39	1.00000	0.00000	1	1.00000	
psaC	-524.8167	40	1.00000	-524.8167	39	1.00000	0.00000	1	1.00000	
psaI	-229.6909	40	1.00000	-229.6909	39	1.00000	0.00000	1	1.00000	
psaJ	-284.4483	40	1.00000	-284.4483	39	1.00000	0.00000	1	1.00000	
psbA	-2059.2479	40	1.00000	-2059.2479	39	1.00000	0.00000	1	1.00000	
psbB	-3248.9049	40	1.00000	-3248.9049	39	1.00000	0.00000	1	1.00000	
psbC	-2800.1976	40	1.00000	-2800.1976	39	1.00000	0.00000	1	1.00000	
psbD	-1912.6536	40	1.00000	-1912.6536	39	1.00000	0.00000	1	1.00000	
psbE	-449.9496	40	1.00000	-449.9496	39	1.00000	0.00000	1	1.00000	
psbH	-606.5578	40	1.00000	-606.5578	39	1.00000	0.00000	1	1.00000	
psbI	-195.1786	40	1.00000	-195.1786	39	1.00000	0.00000	1	1.00000	
psbK	-396.6888	40	1.00000	-396.6888	39	1.00000	0.00000	1	1.00000	
psbT	-209.9403	40	3.61731	-209.9403	39	1.00000	0.00000	1	1.00000	
rbcL	-3037.4388	40	1.00000	-3037.4388	39	1.00000	0.00000	1	1.00000	
rpl14	-738.1688	40	1.00000	-738.1688	39	1.00000	0.00000	1	1.00000	
rpl2	-1432.4256	40	1.00000	-1432.4256	39	1.00000	0.00000	1	1.00000	
rpl16	-839.8404	40	1.00000	-839.8404	39	1.00000	0.00000	1	1.00000	
rpl20	-891.3605	40	1.00000	-891.3605	39	1.00000	0.00000	1	1.00000	
rpl22	-1331.9698	40	1.00000	-1331.9698	39	1.00000	0.00000	1	1.00000	55 L 0.557

rpl23	-410.7523	40	999.00000	-410.9577	39	1.00000	0.41073	1	0.47840	**48 L 0.903***	
rpl33	-481.9047	40	1.00000	-481.9047	39	1.00000	0.00000	1	1.00000		
rpoA	-2781.8015	40	21.57604	-2782.7165	39	1.00000	1.82994	1	0.82386	**243 N 0.799**	243 N 0.850
rpoB	-7411.1338	40	1.00000	-7411.1338	39	1.00000	0.00000	1	1.00000	90 N 0.540 110 F 0.532 113 F 0.542 115 I 0.540 509 T 0.544 556 C 0.558 571 V 0.535 674 N 0.537 876 N 0.540 906 Q 0.559	
rpoC1	-4615.7283	40	1.00000	-4615.7283	39	1.00000	0.00000	1	1.00000		
rpoC2	-11048.2177	40	1.00000	-11048.2177	39	1.00000	0.00000	1	1.00000	623 E 0.508 730 N 0.514 1040 I 0.502	
rps11	-895.5171	40	1.00000	-895.5171	39	1.00000	0.00000	1	1.00000		
rps14	-644.7703	40	1.00000	-644.7703	39	1.00000	0.00000	1	1.00000		
rps15	-758.5680	40	1.00000	-758.5680	39	1.00000	0.00000	1	1.00000	7 V 0.673 42 F 0.689	
rps16	-545.1351	40	1.00000	-545.1351	39	1.00000	0.00000	1	1.00000		
rps19	-590.1963	40	1.00000	-590.1963	39	1.00000	0.00000	1	1.00000		
rps2	-1629.2142	40	1.00000	-1629.2142	39	1.00000	0.00000	1	1.00000		
rps3	-1932.6664	40	1.00000	-1932.6664	39	1.00000	0.00000	1	1.00000	51 R 0.552 89 Q 0.513	
rps4	-1466.1588	40	999.00000	-1467.4493	39	1.00000	2.58099	1	0.89185	**149 M 0.785**	**149 M 0.932***
rps7	-718.3846	40	1.00000	-718.3846	39	1.00000	0.00000	1	1.00000		
rps8	-930.6324	40	1.00000	-930.6324	39	1.00000	0.00000	1	1.00000	75 M 0.533 112 L 0.550	
ycf1	-17791.7036	40	93.93344	-17793.1402	39	1.00000	2.87323	1	0.90994		
ycf2	-11723.8839	40	1.00000	-11723.8839	39	1.00000	0.00000	1	1.00000		
ycf3	-930.5433	40	1.00000	-930.5433	39	1.00000	0.00000	1	1.00000		
ycf4	-1317.1924	40	1.00000	-1317.1985	39	1.00000	0.01230	1	0.08832	**19 A 0.703 23F 0.718 159 Q 0.710**	

*: $P>0.900$, **: $P>0.950$, Bold font: $P>0.700$

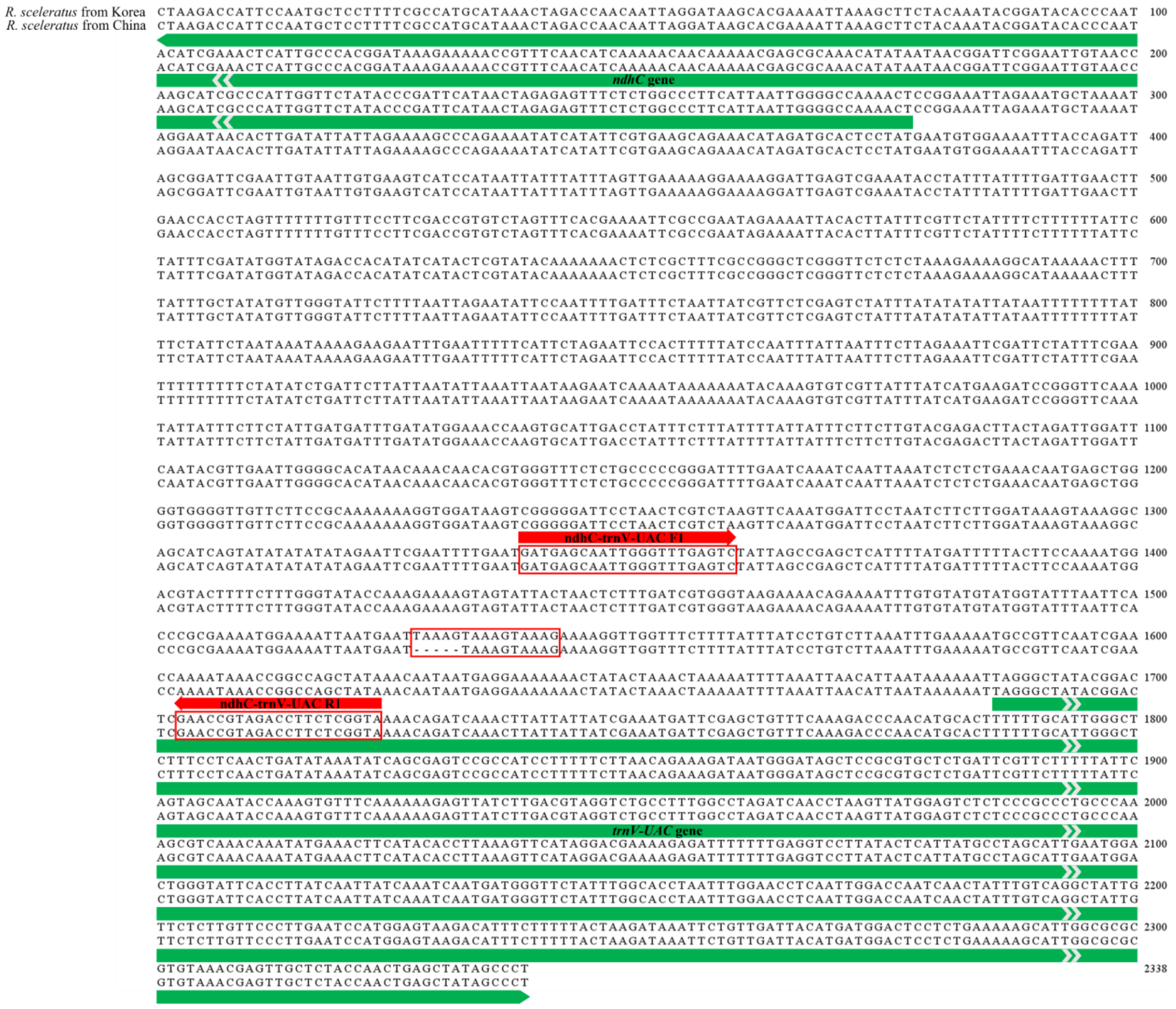

Figure S1. The alignment of the chloroplast sequence was based on the chloroplast genome of *R. sceleratus* from Korea and China. Forward

primer: 5'-GATGAGCAATTGGGTTTGAGTC-3', reverse primer: 5'-TACCGAGAAGGTCTACGGTTC-3', product size: 386 bp.

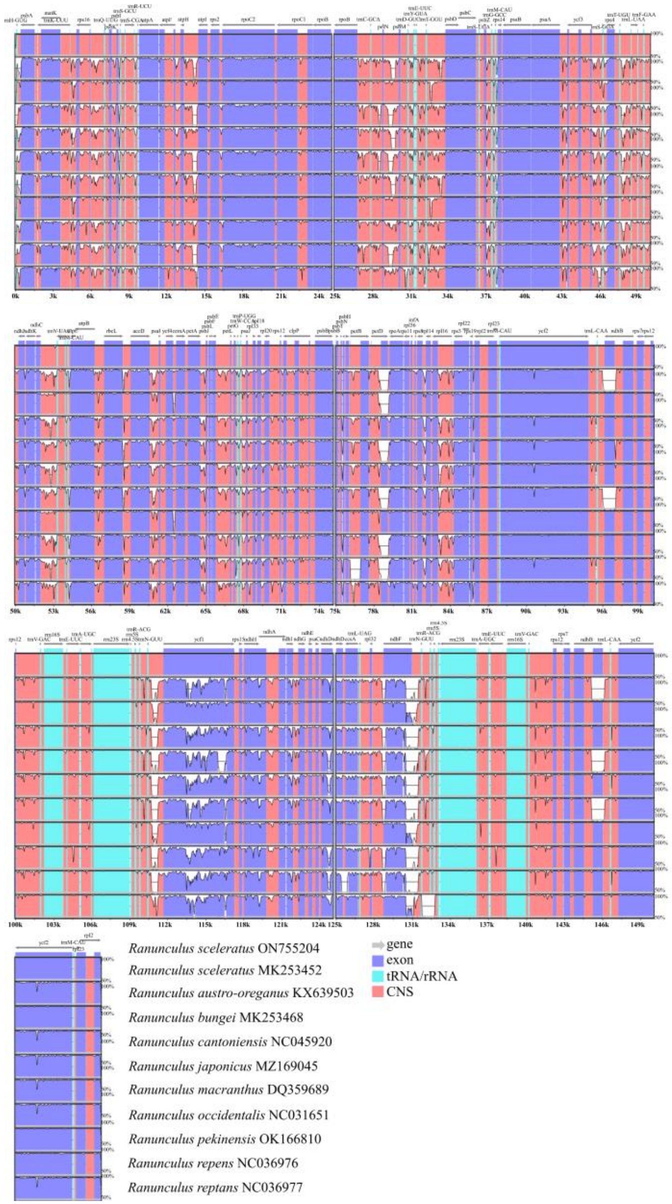

Ranunculus sceleratus ON755204
Ranunculus sceleratus MK253452
Ranunculus austro-oreganus KX639503
Ranunculus bungei MK253468
Ranunculus cantoniensis NC045920
Ranunculus japonicus MZ169045
Ranunculus macranthus DQ359689
Ranunculus occidentalis NC031651
Ranunculus pekinensis OK166810
Ranunculus repens NC036976
Ranunculus reptans NC036977
gene
exon
tRNA/rRNA
CNS

Figure S2. The alignment of the chloroplast sequence was based on the chloroplast genome of *R. sceleratus* from Korea.

Figure S3. Maximum Likelihood and Bayesian Inference phylogenetic tree of specific barcode regions markers.

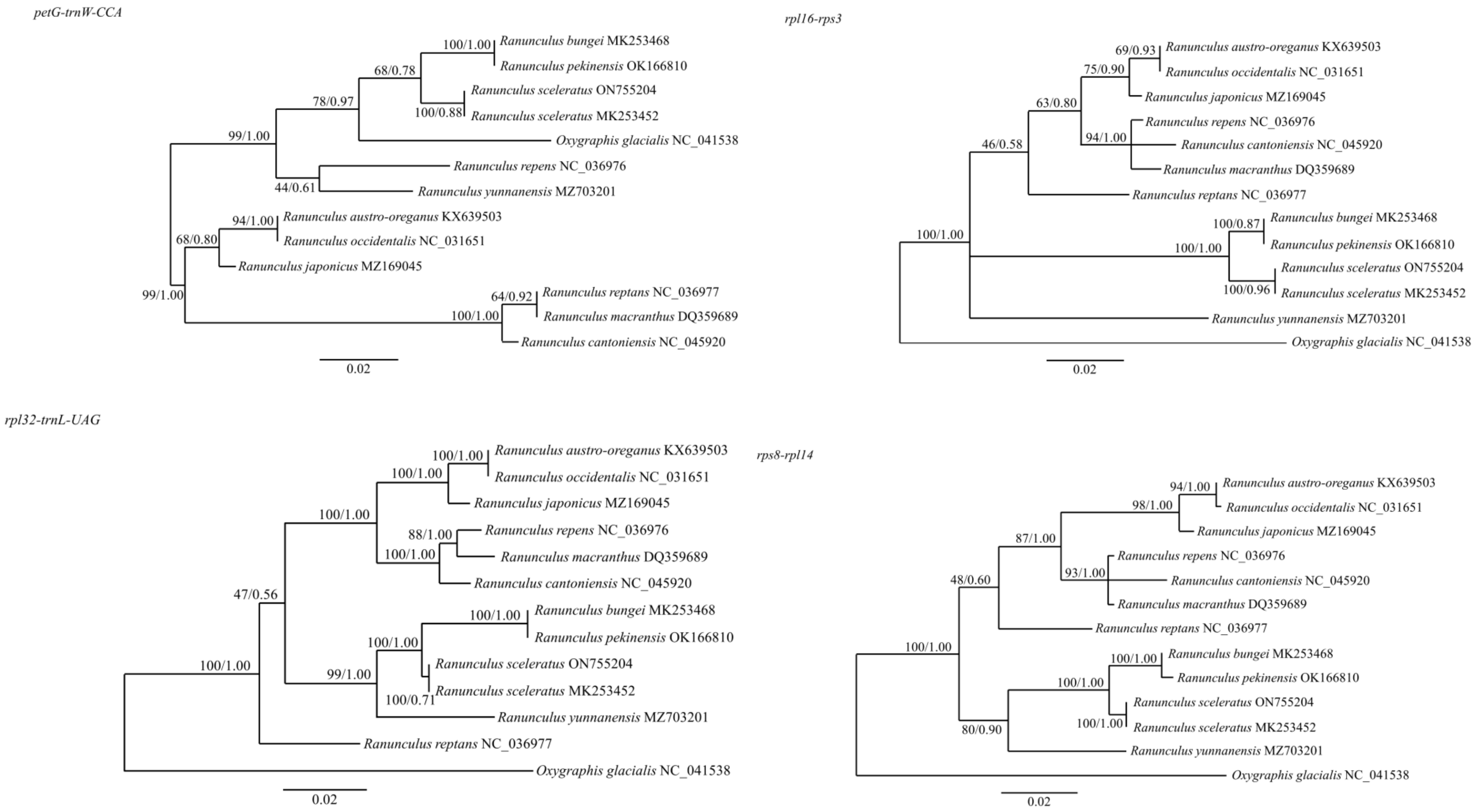

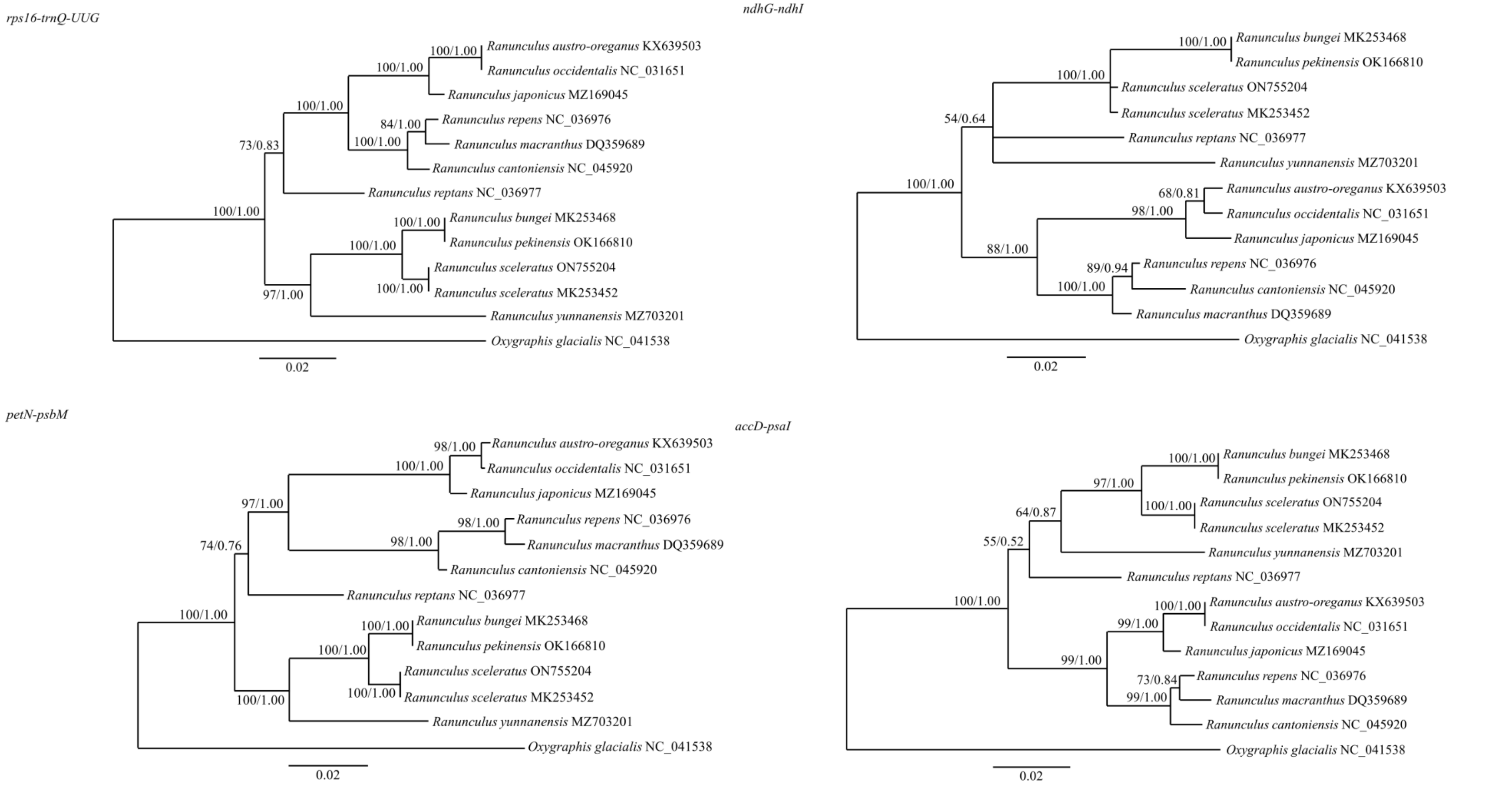
rps16-trnQ-UUG
100/1.00
Ranunculus austro-oreganus KX639503
Ranunculus occidentalis NC_031651
Ranunculus japonicus MZ169045
84/1.00
Ranunculus repens NC_036976
Ranunculus macranthus DQ359689
Ranunculus cantoniensis NC_045920
Ranunculus reptans NC_036977
73/0.83
Ranunculus bungei MK253468
Ranunculus pekinensis OK166810
Ranunculus sceleratus ON755204
Ranunculus sceleratus MK253452
97/1.00
Ranunculus yunnanensis MZ703201
Oxygraphis glacialis NC_041538
0.02
ndhG-ndhI
Ranunculus bungei MK253468
Ranunculus pekinensis OK166810
Ranunculus sceleratus ON755204
Ranunculus sceleratus MK253452
54/0.64
Ranunculus reptans NC_036977
Ranunculus yunnanensis MZ703201
68/0.81
Ranunculus austro-oreganus KX639503
Ranunculus occidentalis NC_031651
98/1.00
Ranunculus japonicus MZ169045
88/1.00
89/0.94
Ranunculus repens NC_036976
Ranunculus cantoniensis NC_045920
Ranunculus macranthus DQ359689
Oxygraphis glacialis NC_041538
0.02
petN-psbM
98/1.00
Ranunculus austro-oreganus KX639503
Ranunculus occidentalis NC_031651
97/1.00
Ranunculus japonicus MZ169045
98/1.00
Ranunculus repens NC_036976
Ranunculus macranthus DQ359689
74/0.76
Ranunculus cantoniensis NC_045920
Ranunculus reptans NC_036977
Ranunculus bungei MK253468
Ranunculus pekinensis OK166810
Ranunculus sceleratus ON755204
Ranunculus sceleratus MK253452
Ranunculus yunnanensis MZ703201
Oxygraphis glacialis NC_041538
0.02
accD-psaI
Ranunculus bungei MK253468
97/1.00
Ranunculus pekinensis OK166810
64/0.87
Ranunculus sceleratus ON755204
55/0.52
Ranunculus sceleratus MK253452
Ranunculus yunnanensis MZ703201
Ranunculus reptans NC_036977
Ranunculus austro-oreganus KX639503
99/1.00
Ranunculus occidentalis NC_031651
Ranunculus japonicus MZ169045
73/0.84
Ranunculus repens NC_036976
Ranunculus macranthus DQ359689
Ranunculus cantoniensis NC_045920
Oxygraphis glacialis NC_041538
0.02

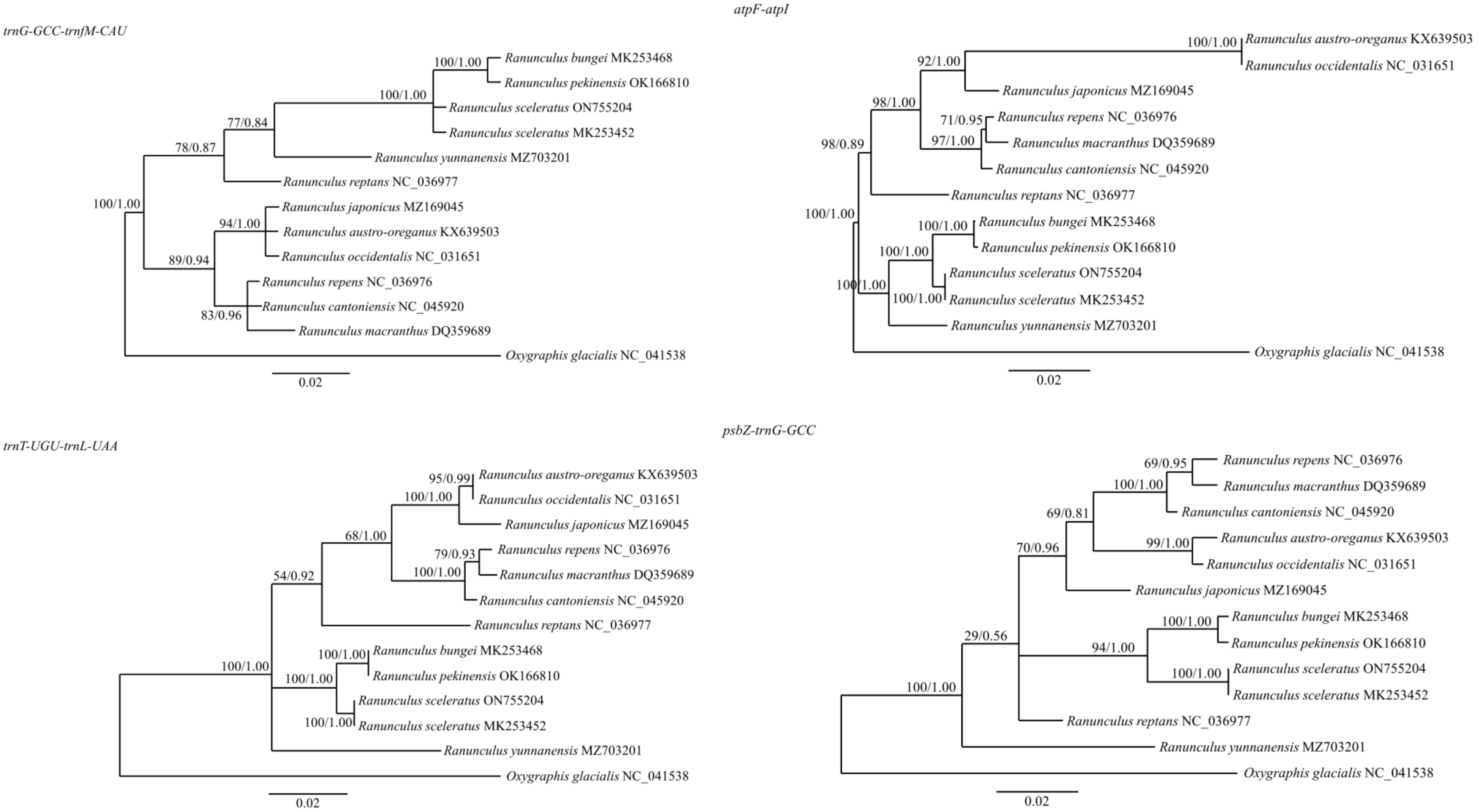
trnG-GCC-trnfM-CAU
100/1.00
78/0.87
77/0.84
100/1.00
100/1.00
Ranunculus bungei MK253468
Ranunculus pekinensis OK166810
Ranunculus sceleratus ON755204
Ranunculus sceleratus MK253452
Ranunculus yunnanensis MZ703201
Ranunculus reptans NC_036977
89/0.94
94/1.00
Ranunculus japonicus MZ169045
Ranunculus austro-oreganus KX639503
Ranunculus occidentalis NC_031651
83/0.96
Ranunculus repens NC_036976
Ranunculus cantoniensis NC_045920
Ranunculus macranthus DQ359689
Oxygraphis glacialis NC_041538
0.02
atpF-atpI
100/1.00
98/0.89
98/1.00
92/1.00
100/1.00
Ranunculus austro-oreganus KX639503
Ranunculus occidentalis NC_031651
Ranunculus japonicus MZ169045
97/1.00
71/0.95
Ranunculus repens NC_036976
Ranunculus macranthus DQ359689
Ranunculus cantoniensis NC_045920
Ranunculus reptans NC_036977
100/1.00
100/1.00
100/1.00
Ranunculus bungei MK253468
Ranunculus pekinensis OK166810
100/1.00
Ranunculus sceleratus ON755204
Ranunculus sceleratus MK253452
Ranunculus yunnanensis MZ703201
Oxygraphis glacialis NC_041538
0.02
trnT-UGU-trnL-UAA
100/1.00
54/0.92
68/1.00
100/1.00
95/0.99
Ranunculus austro-oreganus KX639503
Ranunculus occidentalis NC_031651
Ranunculus japonicus MZ169045
100/1.00
79/0.93
Ranunculus repens NC_036976
Ranunculus macranthus DQ359689
Ranunculus cantoniensis NC_045920
Ranunculus reptans NC_036977
100/1.00
100/1.00
Ranunculus bungei MK253468
Ranunculus pekinensis OK166810
100/1.00
Ranunculus sceleratus ON755204
Ranunculus sceleratus MK253452
Ranunculus yunnanensis MZ703201
Oxygraphis glacialis NC_041538
0.02
psbZ-trnG-GCC
100/1.00
29/0.56
70/0.96
69/0.81
100/1.00
69/0.95
Ranunculus repens NC_036976
Ranunculus macranthus DQ359689
Ranunculus cantoniensis NC_045920
99/1.00
Ranunculus austro-oreganus KX639503
Ranunculus occidentalis NC_031651
Ranunculus japonicus MZ169045
94/1.00
100/1.00
Ranunculus bungei MK253468
Ranunculus pekinensis OK166810
100/1.00
Ranunculus sceleratus ON755204
Ranunculus sceleratus MK253452
Ranunculus reptans NC_036977
Ranunculus yunnanensis MZ703201
Oxygraphis glacialis NC_041538
0.02

trnK-UUU-rps16

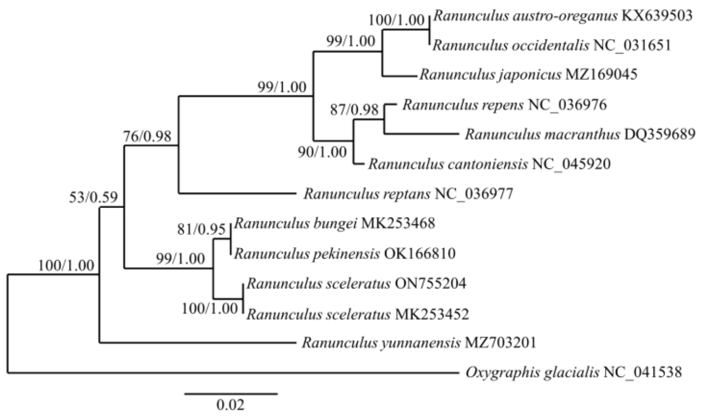

ndhC-trnV-UAC

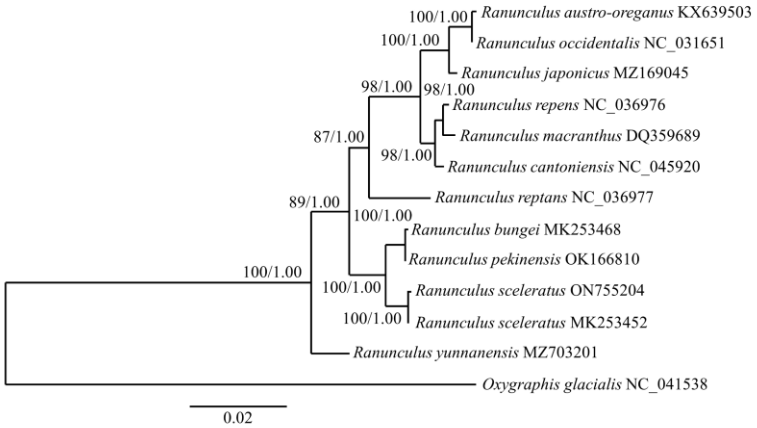

trnT-GGU-psbD

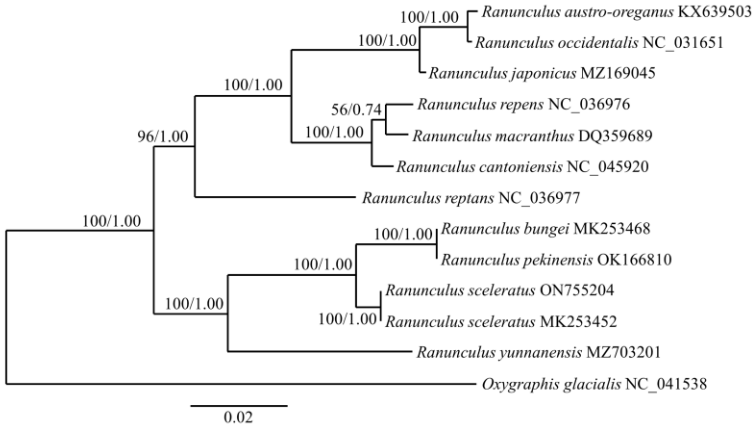

psbE-petL

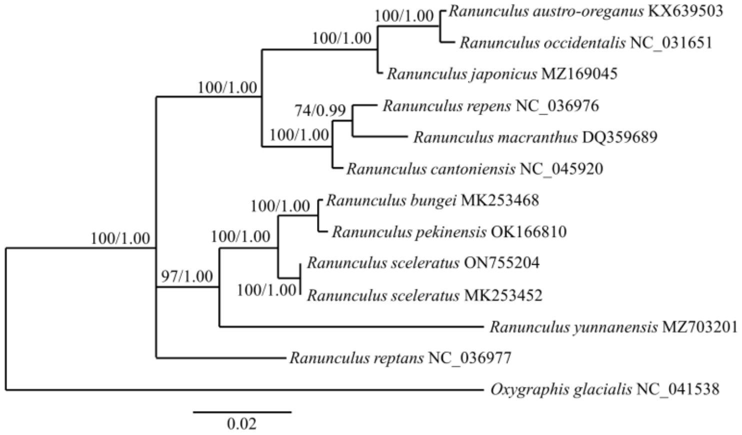

Figure S4. Maximum Likelihood and Bayesian Inference phylogenetic trees of combination markers of intergenic regions.

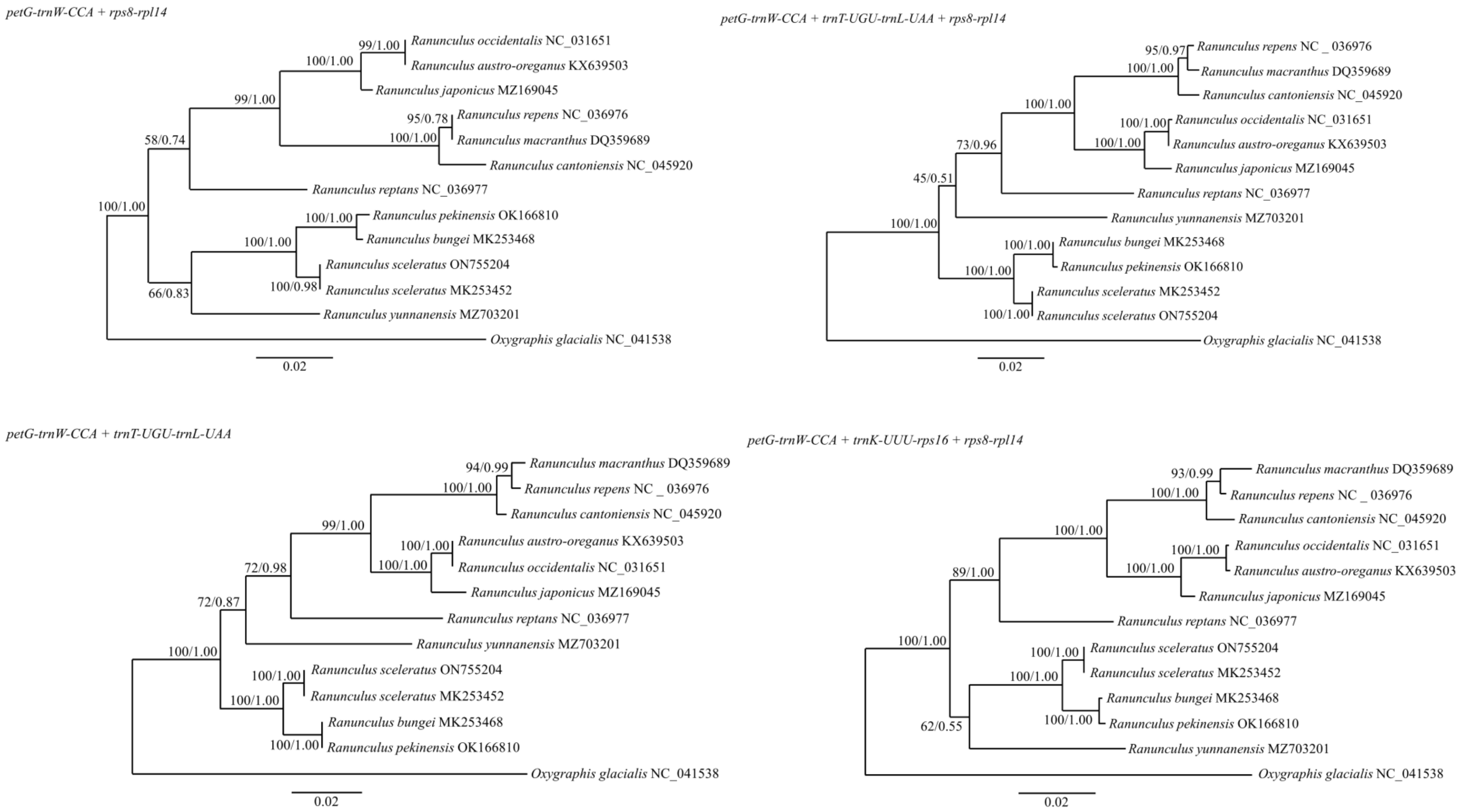

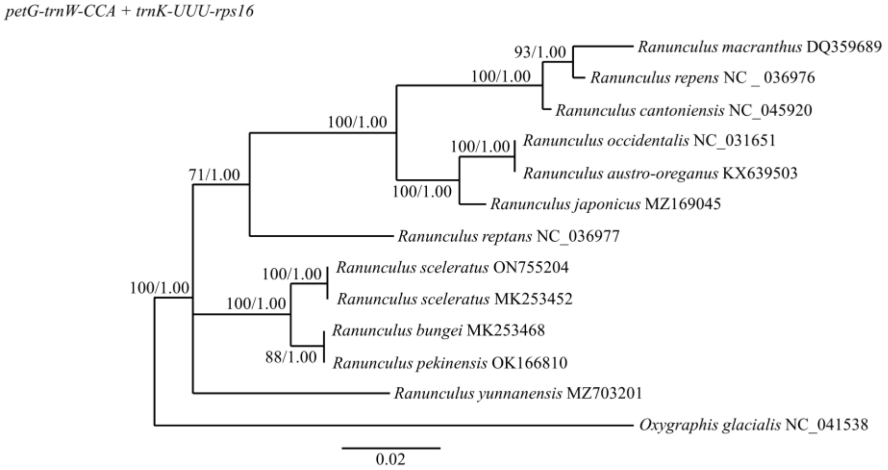

petG-trnW-CCA + trnK-UUU-rps16
Ranunculus macranthus DQ359689
Ranunculus repens NC _ 036976
Ranunculus cantoniensis NC_045920
Ranunculus occidentalis NC_031651
Ranunculus austro-oreganus KX639503
Ranunculus japonicus MZ169045
Ranunculus reptans NC_036977
Ranunculus sceleratus ON755204
Ranunculus sceleratus MK253452
Ranunculus bungei MK253468
Ranunculus pekinensis OK166810
Ranunculus yunnanensis MZ703201
Oxygraphis glacialis NC_041538
93/1.00
100/1.00
100/1.00
100/1.00
100/1.00
71/1.00
100/1.00
100/1.00
100/1.00
88/1.00
0.02

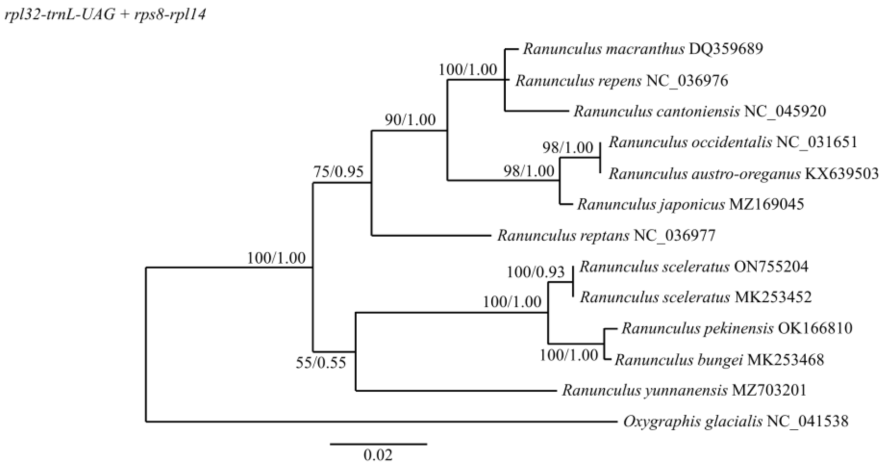

rpl32-trnL-UAG + rps8-rpl14
Ranunculus macranthus DQ359689
Ranunculus repens NC_036976
Ranunculus cantoniensis NC_045920
Ranunculus occidentalis NC_031651
Ranunculus austro-oreganus KX639503
Ranunculus japonicus MZ169045
Ranunculus reptans NC_036977
Ranunculus sceleratus ON755204
Ranunculus sceleratus MK253452
Ranunculus pekinensis OK166810
Ranunculus bungei MK253468
Ranunculus yunnanensis MZ703201
Oxygraphis glacialis NC_041538
100/1.00
90/1.00
98/1.00
98/1.00
75/0.95
100/1.00
100/0.93
100/1.00
100/1.00
55/0.55
0.02

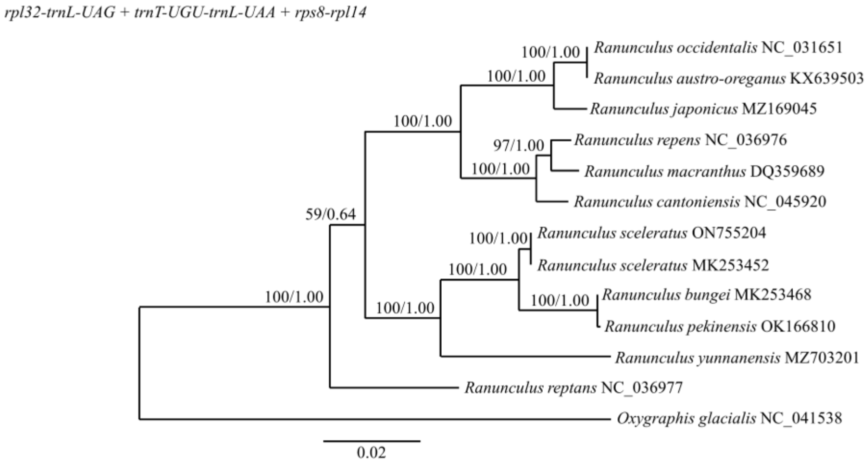

rpl32-trnL-UAG + trnT-UGU-trnL-UAA + rps8-rpl14
Ranunculus occidentalis NC_031651
Ranunculus austro-oreganus KX639503
Ranunculus japonicus MZ169045
Ranunculus repens NC_036976
Ranunculus macranthus DQ359689
Ranunculus cantoniensis NC_045920
Ranunculus sceleratus ON755204
Ranunculus sceleratus MK253452
Ranunculus bungei MK253468
Ranunculus pekinensis OK166810
Ranunculus yunnanensis MZ703201
Ranunculus reptans NC_036977
Oxygraphis glacialis NC_041538
100/1.00
100/1.00
100/1.00
97/1.00
100/1.00
59/0.64
100/1.00
100/1.00
100/1.00
100/1.00
100/1.00
0.02

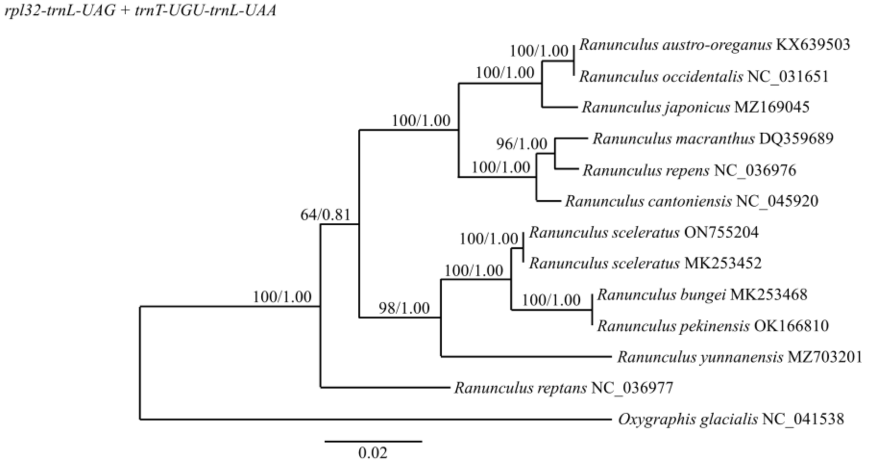

rpl32-trnL-UAG + trnT-UGU-trnL-UAA
Ranunculus austro-oreganus KX639503
Ranunculus occidentalis NC_031651
Ranunculus japonicus MZ169045
Ranunculus macranthus DQ359689
Ranunculus repens NC_036976
Ranunculus cantoniensis NC_045920
Ranunculus sceleratus ON755204
Ranunculus sceleratus MK253452
Ranunculus bungei MK253468
Ranunculus pekinensis OK166810
Ranunculus yunnanensis MZ703201
Ranunculus reptans NC_036977
Oxygraphis glacialis NC_041538
100/1.00
100/1.00
100/1.00
96/1.00
100/1.00
64/0.81
100/1.00
100/1.00
100/1.00
98/1.00
100/1.00
0.02

rpl32-trnL-UAG + trnK-UUU-rps16 + rps8-rpl14

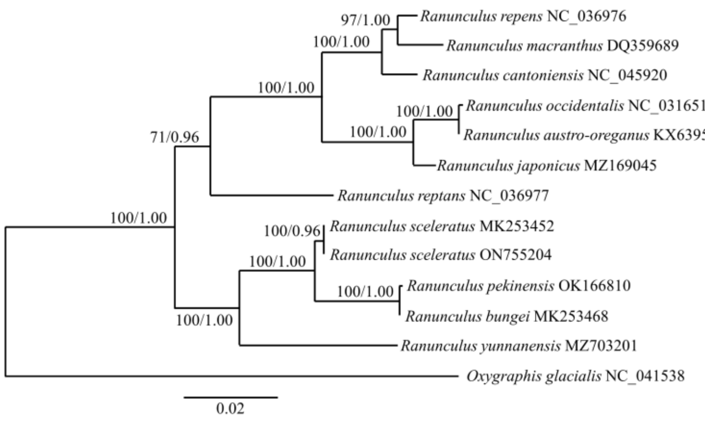

rpl32-trnL-UAG + trnK-UUU-rps16

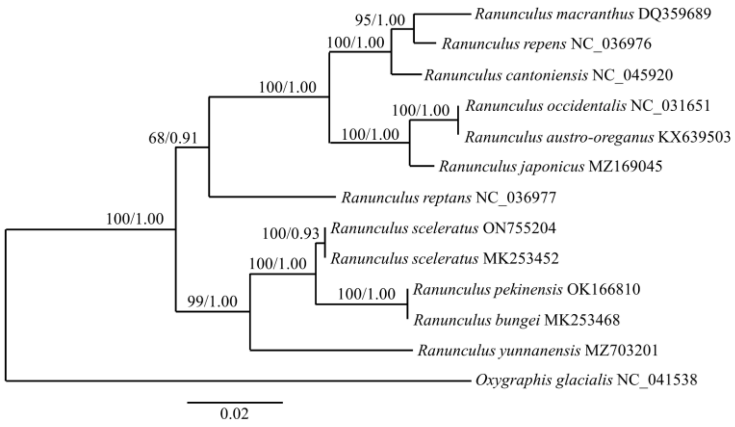

rpl16-rps3 + rps8-rpl14

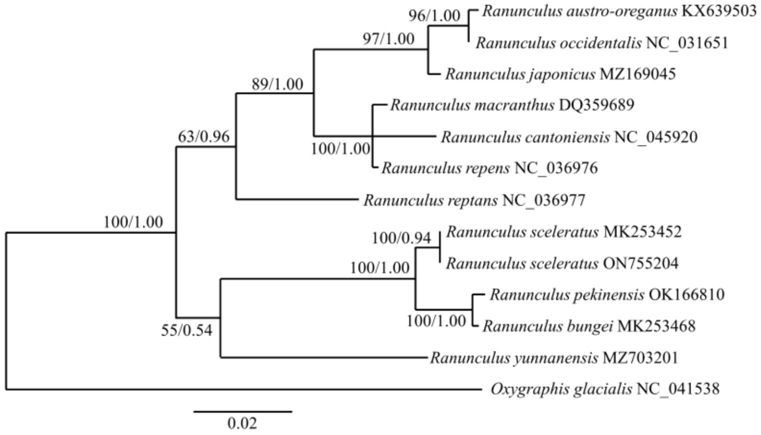

rpl16-rps3 + trnT-UGU-trnL-UAA + rps8-rpl14

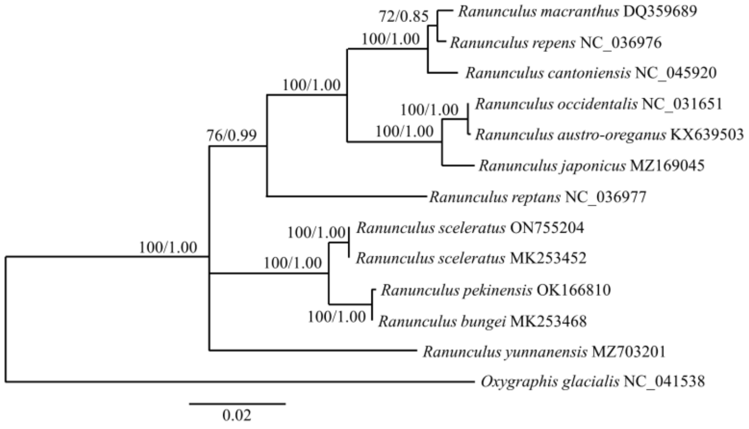

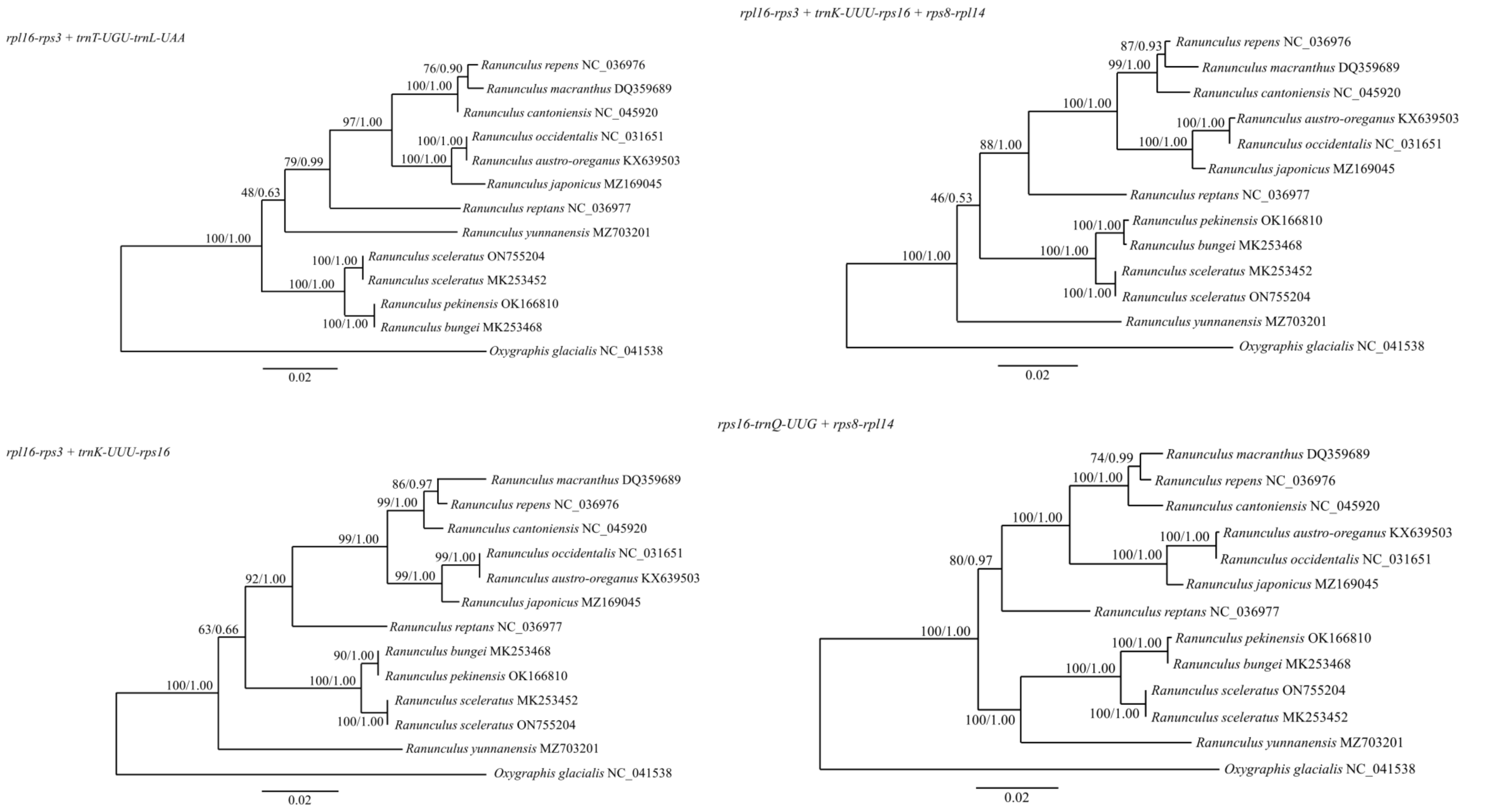
rpl16-rps3 + trnT-UGU-trnL-UAA
Ranunculus repens NC_036976
Ranunculus macranthus DQ359689
Ranunculus cantoniensis NC_045920
Ranunculus occidentalis NC_031651
Ranunculus austro-oreganus KX639503
Ranunculus japonicus MZ169045
Ranunculus reptans NC_036977
Ranunculus yunnanensis MZ703201
Ranunculus sceleratus ON755204
Ranunculus sceleratus MK253452
Ranunculus pekinensis OK166810
Ranunculus bungei MK253468
Oxygraphis glacialis NC_041538
76/0.90
100/1.00
97/1.00
100/1.00
100/1.00
79/0.99
48/0.63
100/1.00
100/1.00
100/1.00
100/1.00
0.02
rpl16-rps3 + trnK-UUU-rps16 + rps8-rpl14
Ranunculus repens NC_036976
Ranunculus macranthus DQ359689
Ranunculus cantoniensis NC_045920
Ranunculus austro-oreganus KX639503
Ranunculus occidentalis NC_031651
Ranunculus japonicus MZ169045
Ranunculus reptans NC_036977
Ranunculus pekinensis OK166810
Ranunculus bungei MK253468
Ranunculus sceleratus MK253452
Ranunculus sceleratus ON755204
Ranunculus yunnanensis MZ703201
Oxygraphis glacialis NC_041538
87/0.93
99/1.00
100/1.00
100/1.00
100/1.00
88/1.00
46/0.53
100/1.00
100/1.00
100/1.00
100/1.00
0.02
rpl16-rps3 + trnK-UUU-rps16
Ranunculus macranthus DQ359689
Ranunculus repens NC_036976
Ranunculus cantoniensis NC_045920
Ranunculus occidentalis NC_031651
Ranunculus austro-oreganus KX639503
Ranunculus japonicus MZ169045
Ranunculus reptans NC_036977
Ranunculus bungei MK253468
Ranunculus pekinensis OK166810
Ranunculus sceleratus MK253452
Ranunculus sceleratus ON755204
Ranunculus yunnanensis MZ703201
Oxygraphis glacialis NC_041538
86/0.97
99/1.00
99/1.00
99/1.00
99/1.00
92/1.00
63/0.66
90/1.00
100/1.00
100/1.00
100/1.00
0.02
rps16-trnQ-UUG + rps8-rpl14
Ranunculus macranthus DQ359689
Ranunculus repens NC_036976
Ranunculus cantoniensis NC_045920
Ranunculus austro-oreganus KX639503
Ranunculus occidentalis NC_031651
Ranunculus japonicus MZ169045
Ranunculus reptans NC_036977
Ranunculus pekinensis OK166810
Ranunculus bungei MK253468
Ranunculus sceleratus ON755204
Ranunculus sceleratus MK253452
Ranunculus yunnanensis MZ703201
Oxygraphis glacialis NC_041538
74/0.99
100/1.00
100/1.00
100/1.00
100/1.00
80/0.97
100/1.00
100/1.00
100/1.00
100/1.00
100/1.00
0.02

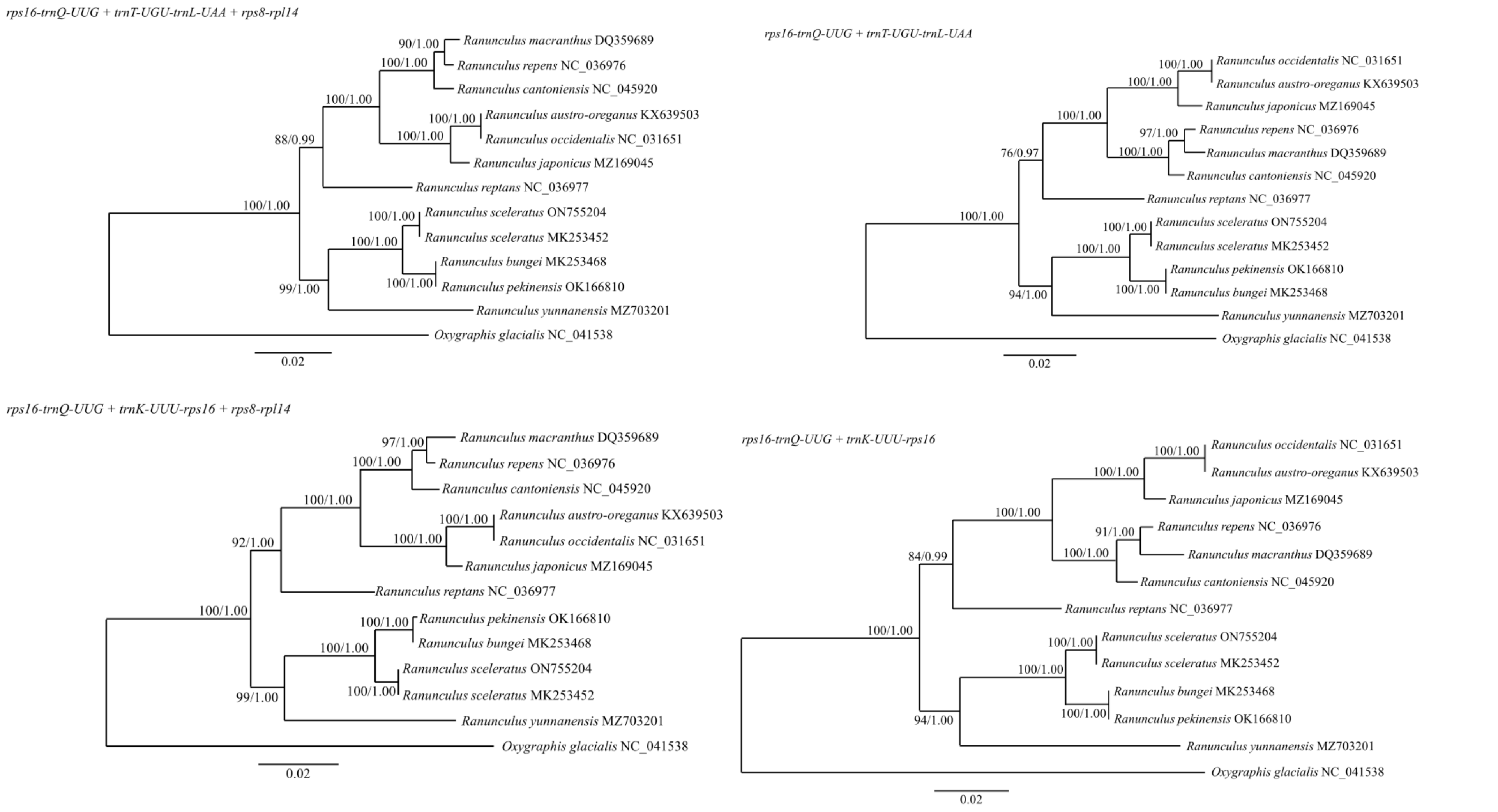

rps16-trnQ-UUG + trnT-UGU-trnL-UAA + rps8-rpl14
90/1.00
100/1.00
100/1.00
88/0.99
100/1.00
100/1.00
100/1.00
100/1.00
100/1.00
99/1.00
100/1.00
Ranunculus macranthus DQ359689
Ranunculus repens NC_036976
Ranunculus cantoniensis NC_045920
Ranunculus austro-oreganus KX639503
Ranunculus occidentalis NC_031651
Ranunculus japonicus MZ169045
Ranunculus reptans NC_036977
Ranunculus sceleratus ON755204
Ranunculus sceleratus MK253452
Ranunculus bungei MK253468
Ranunculus pekinensis OK166810
Ranunculus yunnanensis MZ703201
Oxygraphis glacialis NC_041538
0.02
rps16-trnQ-UUG + trnT-UGU-trnL-UAA
100/1.00
100/1.00
100/1.00
97/1.00
100/1.00
76/0.97
100/1.00
100/1.00
100/1.00
100/1.00
94/1.00
Ranunculus occidentalis NC_031651
Ranunculus austro-oreganus KX639503
Ranunculus japonicus MZ169045
Ranunculus repens NC_036976
Ranunculus macranthus DQ359689
Ranunculus cantoniensis NC_045920
Ranunculus reptans NC_036977
Ranunculus sceleratus ON755204
Ranunculus sceleratus MK253452
Ranunculus pekinensis OK166810
Ranunculus bungei MK253468
Ranunculus yunnanensis MZ703201
Oxygraphis glacialis NC_041538
0.02
rps16-trnQ-UUG + trnK-UUU-rps16 + rps8-rpl14
97/1.00
100/1.00
100/1.00
100/1.00
92/1.00
100/1.00
100/1.00
100/1.00
100/1.00
100/1.00
99/1.00
Ranunculus macranthus DQ359689
Ranunculus repens NC_036976
Ranunculus cantoniensis NC_045920
Ranunculus austro-oreganus KX639503
Ranunculus occidentalis NC_031651
Ranunculus japonicus MZ169045
Ranunculus reptans NC_036977
Ranunculus pekinensis OK166810
Ranunculus bungei MK253468
Ranunculus sceleratus ON755204
Ranunculus sceleratus MK253452
Ranunculus yunnanensis MZ703201
Oxygraphis glacialis NC_041538
0.02
rps16-trnQ-UUG + trnK-UUU-rps16
100/1.00
100/1.00
100/1.00
91/1.00
100/1.00
84/0.99
100/1.00
100/1.00
100/1.00
100/1.00
94/1.00
Ranunculus occidentalis NC_031651
Ranunculus austro-oreganus KX639503
Ranunculus japonicus MZ169045
Ranunculus repens NC_036976
Ranunculus macranthus DQ359689
Ranunculus cantoniensis NC_045920
Ranunculus reptans NC_036977
Ranunculus sceleratus ON755204
Ranunculus sceleratus MK253452
Ranunculus bungei MK253468
Ranunculus pekinensis OK166810
Ranunculus yunnanensis MZ703201
Oxygraphis glacialis NC_041538
0.02

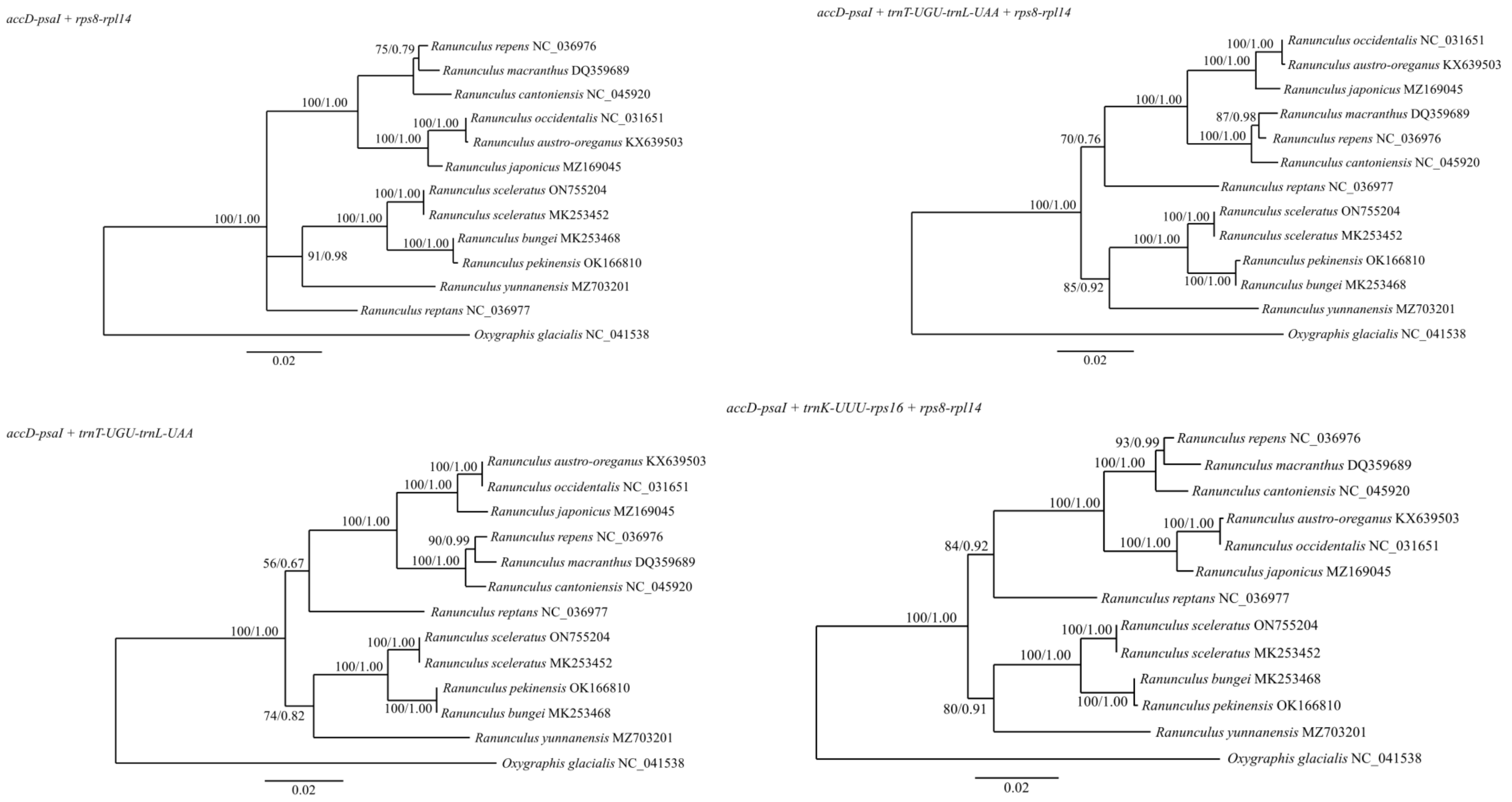

accD-psaI + rps8-rpl14
75/0.79
Ranunculus repens NC_036976
Ranunculus macranthus DQ359689
Ranunculus cantoniensis NC_045920
100/1.00
Ranunculus occidentalis NC_031651
Ranunculus austro-oreganus KX639503
Ranunculus japonicus MZ169045
Ranunculus sceleratus ON755204
Ranunculus sceleratus MK253452
Ranunculus bungei MK253468
Ranunculus pekinensis OK166810
91/0.98
Ranunculus yunnanensis MZ703201
Ranunculus reptans NC_036977
Oxygraphis glacialis NC_041538
0.02
accD-psaI + trnT-UGU-trnL-UAA + rps8-rpl14
87/0.98
70/0.76
85/0.92
accD-psaI + trnT-UGU-trnL-UAA
90/0.99
56/0.67
74/0.82
accD-psaI + trnK-UUU-rps16 + rps8-rpl14
93/0.99
84/0.92
80/0.91

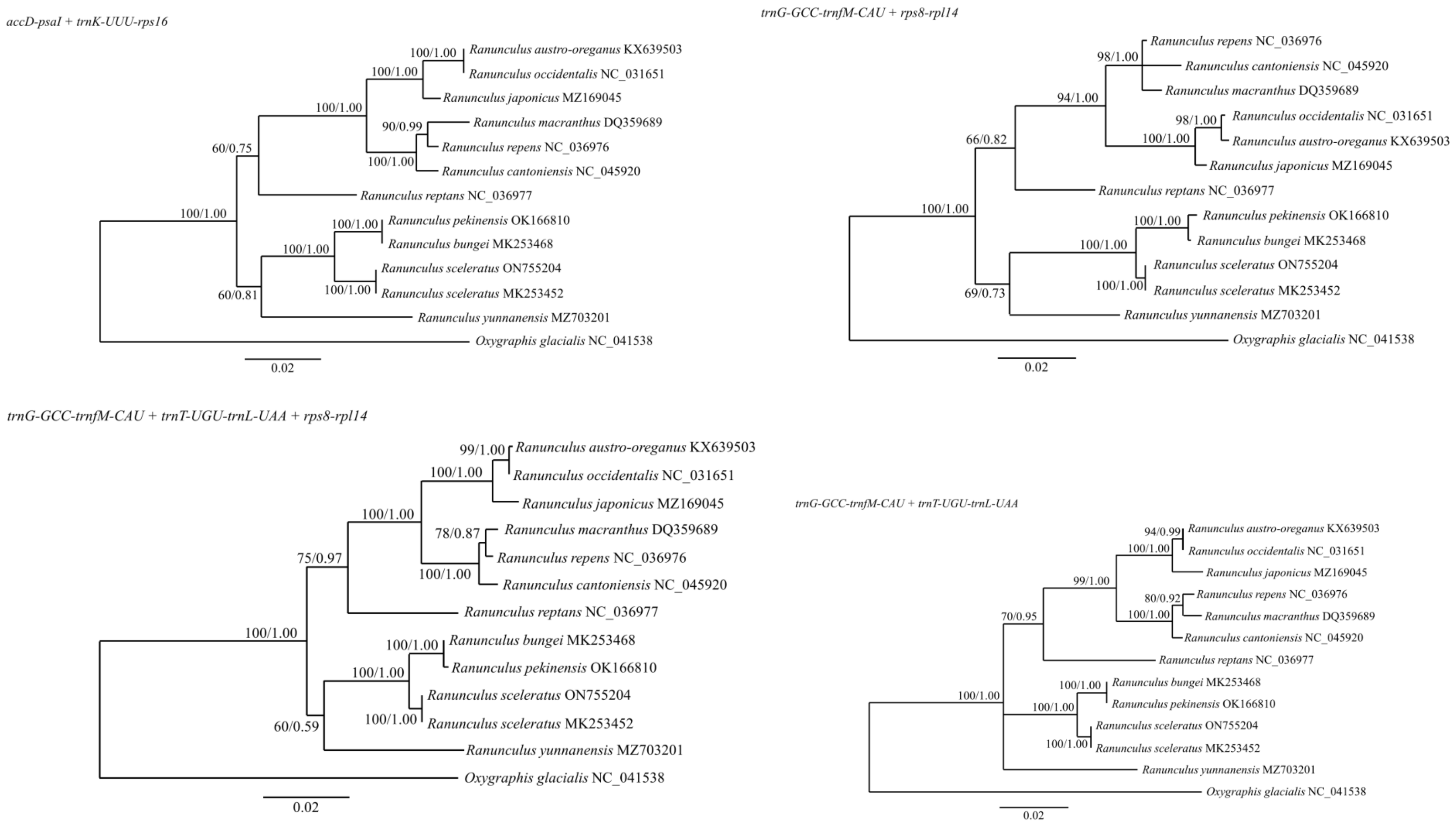
accD-psaI + trnK-UUU-rps16
Ranunculus austro-oreganus KX639503
Ranunculus occidentalis NC_031651
Ranunculus japonicus MZ169045
Ranunculus macranthus DQ359689
Ranunculus repens NC_036976
Ranunculus cantoniensis NC_045920
Ranunculus reptans NC_036977
Ranunculus pekinensis OK166810
Ranunculus bungei MK253468
Ranunculus sceleratus ON755204
Ranunculus sceleratus MK253452
Ranunculus yunnanensis MZ703201
Oxygraphis glacialis NC_041538
100/1.00
100/1.00
100/1.00
90/0.99
100/1.00
60/0.75
100/1.00
100/1.00
100/1.00
100/1.00
60/0.81
0.02
trnG-GCC-trnfM-CAU + rps8-rpl14
Ranunculus repens NC_036976
Ranunculus cantoniensis NC_045920
Ranunculus macranthus DQ359689
Ranunculus occidentalis NC_031651
Ranunculus austro-oreganus KX639503
Ranunculus japonicus MZ169045
Ranunculus reptans NC_036977
Ranunculus pekinensis OK166810
Ranunculus bungei MK253468
Ranunculus sceleratus ON755204
Ranunculus sceleratus MK253452
Ranunculus yunnanensis MZ703201
Oxygraphis glacialis NC_041538
98/1.00
94/1.00
98/1.00
100/1.00
66/0.82
100/1.00
100/1.00
100/1.00
100/1.00
69/0.73
0.02
trnG-GCC-trnfM-CAU + trnT-UGU-trnL-UAA + rps8-rpl14
Ranunculus austro-oreganus KX639503
Ranunculus occidentalis NC_031651
Ranunculus japonicus MZ169045
Ranunculus macranthus DQ359689
Ranunculus repens NC_036976
Ranunculus cantoniensis NC_045920
Ranunculus reptans NC_036977
Ranunculus bungei MK253468
Ranunculus pekinensis OK166810
Ranunculus sceleratus ON755204
Ranunculus sceleratus MK253452
Ranunculus yunnanensis MZ703201
Oxygraphis glacialis NC_041538
99/1.00
100/1.00
100/1.00
78/0.87
100/1.00
75/0.97
100/1.00
100/1.00
100/1.00
100/1.00
60/0.59
0.02
trnG-GCC-trnfM-CAU + trnT-UGU-trnL-UAA
Ranunculus austro-oreganus KX639503
Ranunculus occidentalis NC_031651
Ranunculus japonicus MZ169045
Ranunculus repens NC_036976
Ranunculus macranthus DQ359689
Ranunculus cantoniensis NC_045920
Ranunculus reptans NC_036977
Ranunculus bungei MK253468
Ranunculus pekinensis OK166810
Ranunculus sceleratus ON755204
Ranunculus sceleratus MK253452
Ranunculus yunnanensis MZ703201
Oxygraphis glacialis NC_041538
94/0.99
100/1.00
99/1.00
80/0.92
100/1.00
70/0.95
100/1.00
100/1.00
100/1.00
100/1.00
0.02

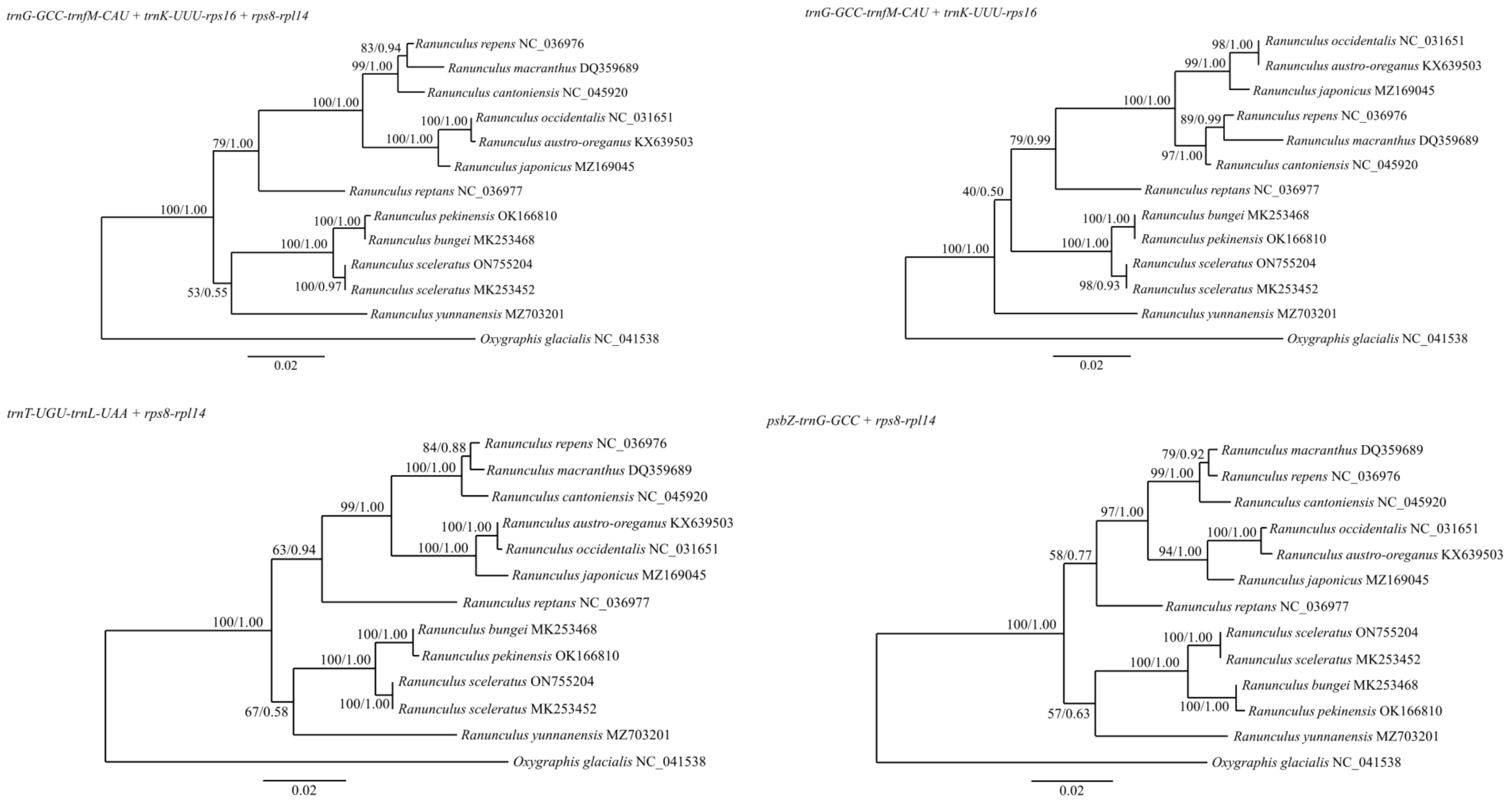

trnG-GCC-trnfM-CAU + trnK-UUU-rps16 + rps8-rpl14
83/0.94
99/1.00
100/1.00
100/1.00
100/1.00
79/1.00
100/1.00
100/1.00
100/1.00
53/0.55
100/0.97
Ranunculus repens NC_036976
Ranunculus macranthus DQ359689
Ranunculus cantoniensis NC_045920
Ranunculus occidentalis NC_031651
Ranunculus austro-oreganus KX639503
Ranunculus japonicus MZ169045
Ranunculus reptans NC_036977
Ranunculus pekinensis OK166810
Ranunculus bungei MK253468
Ranunculus sceleratus ON755204
Ranunculus sceleratus MK253452
Ranunculus yunnanensis MZ703201
Oxygraphis glacialis NC_041538
0.02
trnG-GCC-trnfM-CAU + trnK-UUU-rps16
98/1.00
99/1.00
100/1.00
89/0.99
97/1.00
79/0.99
40/0.50
100/1.00
100/1.00
100/1.00
98/0.93
Ranunculus occidentalis NC_031651
Ranunculus austro-oreganus KX639503
Ranunculus japonicus MZ169045
Ranunculus repens NC_036976
Ranunculus macranthus DQ359689
Ranunculus cantoniensis NC_045920
Ranunculus reptans NC_036977
Ranunculus bungei MK253468
Ranunculus pekinensis OK166810
Ranunculus sceleratus ON755204
Ranunculus sceleratus MK253452
Ranunculus yunnanensis MZ703201
Oxygraphis glacialis NC_041538
0.02
trnT-UGU-trnL-UAA + rps8-rpl14
84/0.88
100/1.00
99/1.00
100/1.00
100/1.00
63/0.94
100/1.00
100/1.00
100/1.00
67/0.58
100/1.00
Ranunculus repens NC_036976
Ranunculus macranthus DQ359689
Ranunculus cantoniensis NC_045920
Ranunculus austro-oreganus KX639503
Ranunculus occidentalis NC_031651
Ranunculus japonicus MZ169045
Ranunculus reptans NC_036977
Ranunculus bungei MK253468
Ranunculus pekinensis OK166810
Ranunculus sceleratus ON755204
Ranunculus sceleratus MK253452
Ranunculus yunnanensis MZ703201
Oxygraphis glacialis NC_041538
0.02
psbZ-trnG-GCC + rps8-rpl14
79/0.92
99/1.00
97/1.00
100/1.00
94/1.00
58/0.77
100/1.00
100/1.00
100/1.00
100/1.00
57/0.63
Ranunculus macranthus DQ359689
Ranunculus repens NC_036976
Ranunculus cantoniensis NC_045920
Ranunculus occidentalis NC_031651
Ranunculus austro-oreganus KX639503
Ranunculus japonicus MZ169045
Ranunculus reptans NC_036977
Ranunculus sceleratus ON755204
Ranunculus sceleratus MK253452
Ranunculus bungei MK253468
Ranunculus pekinensis OK166810
Ranunculus yunnanensis MZ703201
Oxygraphis glacialis NC_041538
0.02

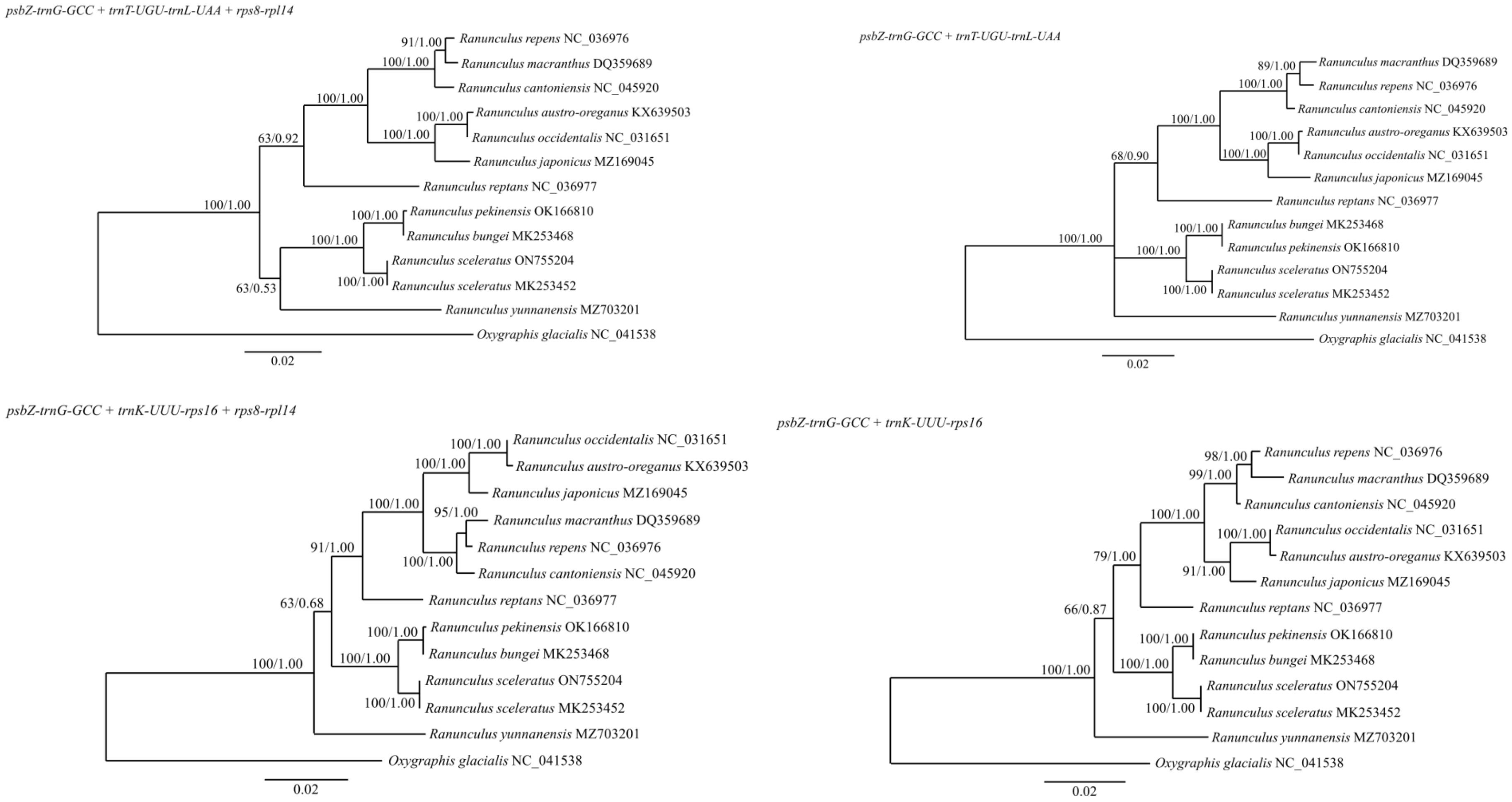

psbZ-trnG-GCC + trnT-UGU-trnL-UAA + rps8-rpl14
91/1.00
100/1.00
100/1.00
63/0.92
100/1.00
100/1.00
100/1.00
100/1.00
100/1.00
63/0.53
100/1.00
Ranunculus repens NC_036976
Ranunculus macranthus DQ359689
Ranunculus cantoniensis NC_045920
Ranunculus austro-oreganus KX639503
Ranunculus occidentalis NC_031651
Ranunculus japonicus MZ169045
Ranunculus reptans NC_036977
Ranunculus pekinensis OK166810
Ranunculus bungei MK253468
Ranunculus sceleratus ON755204
Ranunculus sceleratus MK253452
Ranunculus yunnanensis MZ703201
Oxygraphis glacialis NC_041538
0.02
psbZ-trnG-GCC + trnT-UGU-trnL-UAA
89/1.00
100/1.00
100/1.00
100/1.00
100/1.00
68/0.90
100/1.00
100/1.00
100/1.00
100/1.00
Ranunculus macranthus DQ359689
Ranunculus repens NC_036976
Ranunculus cantoniensis NC_045920
Ranunculus austro-oreganus KX639503
Ranunculus occidentalis NC_031651
Ranunculus japonicus MZ169045
Ranunculus reptans NC_036977
Ranunculus bungei MK253468
Ranunculus pekinensis OK166810
Ranunculus sceleratus ON755204
Ranunculus sceleratus MK253452
Ranunculus yunnanensis MZ703201
Oxygraphis glacialis NC_041538
0.02
psbZ-trnG-GCC + trnK-UUU-rps16 + rps8-rpl14
100/1.00
100/1.00
100/1.00
95/1.00
91/1.00
100/1.00
63/0.68
100/1.00
100/1.00
100/1.00
100/1.00
Ranunculus occidentalis NC_031651
Ranunculus austro-oreganus KX639503
Ranunculus japonicus MZ169045
Ranunculus macranthus DQ359689
Ranunculus repens NC_036976
Ranunculus cantoniensis NC_045920
Ranunculus reptans NC_036977
Ranunculus pekinensis OK166810
Ranunculus bungei MK253468
Ranunculus sceleratus ON755204
Ranunculus sceleratus MK253452
Ranunculus yunnanensis MZ703201
Oxygraphis glacialis NC_041538
0.02
psbZ-trnG-GCC + trnK-UUU-rps16
98/1.00
99/1.00
100/1.00
100/1.00
79/1.00
91/1.00
66/0.87
100/1.00
100/1.00
100/1.00
100/1.00
Ranunculus repens NC_036976
Ranunculus macranthus DQ359689
Ranunculus cantoniensis NC_045920
Ranunculus occidentalis NC_031651
Ranunculus austro-oreganus KX639503
Ranunculus japonicus MZ169045
Ranunculus reptans NC_036977
Ranunculus pekinensis OK166810
Ranunculus bungei MK253468
Ranunculus sceleratus ON755204
Ranunculus sceleratus MK253452
Ranunculus yunnanensis MZ703201
Oxygraphis glacialis NC_041538
0.02

trnK-UUU-rps16 + rps8-rpl14

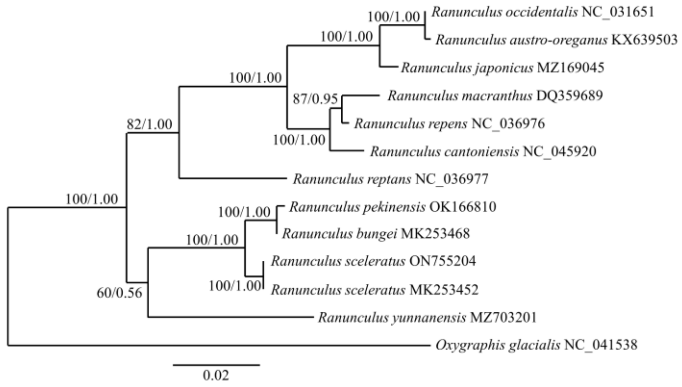

Supplement: Supplementary file 1 [file genes-14-01149-s001.zip › genes-2393207-supplementary.pdf]
